# Mitochondrial-specific perturbation of *Drosophila RNase Z* in neurons leads to motor impairments, disrupted learning and neurodegeneration

**DOI:** 10.1371/journal.pgen.1011938

**Published:** 2025-11-03

**Authors:** Saathvika Rajamani, Lucia Vilchez, Nicole Cracovia, Dritjona Dule, Alessia Vata, Saul Landaverde, Atulya Iyengar, Edward B. Dubrovsky

**Affiliations:** 1 Department of Biological Sciences, Fordham University, New York, New York, United States of America; 2 Department of Biological Sciences, University of Alabama, Tuscaloosa, Alabama, United States of America; 3 Center for Cancer, Genetic Diseases, and Gene Regulation, Fordham University, New York, New York, United States of America; HudsonAlpha Institute for Biotechnology, UNITED STATES OF AMERICA

## Abstract

Clinical studies have linked a rare form of neurological disorder to the highly conserved *RNase Z* gene, which encodes an endoribonuclease responsible for the processing of nuclear and mitochondrial primary tRNA transcripts. Patients harboring mutant variants of this gene exhibit a spectrum of neurological dysfunction; however, no studies to date have established the causality of RNase Z-linked neuropathology. We employed CRISPR/Cas9 technology to create flies with a neuron-specific knockout of the *RNase Z* gene, which is rescued with transgenes encoding a wild-type or a mutant copy of RNase Z. Neuronal activity of RNase Z is vital, as mutants display striking morphological abnormalities in central and peripheral neurons, along with attenuated motor circuit function and associative learning performance. Neuron-specific mutations of RNase Z also led to mitochondrial fragmentation and elevated ROS production. By employing the rescue transgene encoding RNase Z devoid of a mitochondrial targeting signal (MTS), we segregated the mitochondrial activity of RNase Z from that in other compartments, allowing us to assess organelle-specific contributions to disease etiology and progression. We found that mutating mitochondrial RNase Z was sufficient to induce the neuropathology in flies, as they recapitulate the salient phenotypes observed in the pan-neuronal mutants. Collectively, our study validates the pathogenicity of mutant RNase Z and establishes mitochondrial-specific contributions to neuropathology.

## Author summary

Over a third of the world’s population is affected by neurological conditions, making disorders of the nervous system a leading cause of disability. Many neurological disorders have a genetic component, and recent clinical research has linked mutations in genes that process transfer RNA molecules to nervous system dysfunction. One such gene encodes a highly conserved enzyme, RNase Z, involved in the maturation of nuclear and mitochondrial transfer RNAs. Mutations in this gene have been identified in patients with neurological symptoms, but until now, no direct experimental evidence has confirmed its role in causing disease. Using the fruit fly model, we found that flies lacking functional RNase Z in neurons show severe defects in brain structure and behavior, including impaired movement and memory. Importantly, we show that neuronal mitochondria are dysfunctional and that mutating mitochondrial RNase Z is sufficient to produce neurophenotypes. The versatility of the fly model allowed us to study the requirement of this enzyme at an organelle-specific level and provide substantial evidence supporting prior claims classifying RNase Z-linked pathology as a mitochondrial disease. Our findings offer greater insight into how RNase Z mutations contribute to neuropathology in humans and further guide therapeutic development.

## Introduction

Multiple clinical studies have described neurological complications in patients carrying mutant variants of *ELAC2.* This gene encodes an endoribonuclease, RNase Z, involved in transfer RNA (tRNA) maturation [1]. An initial report by Haack and colleagues [2] identified an infant boy compound heterozygous for *ELAC2* alleles (Arg211Stop; Thr520Ile) and presenting with symptoms of neurological impairment and cardiomyopathy. To date, multiple cases of neuropathology have been associated with over 11 recessive alleles of *ELAC2*. Individuals carrying a combination of these alleles show varying levels of microcephaly, muscular hypotonia, loss of sensorineural hearing and motor skills, intellectual disability, and seizures, symptoms consistent with neurodegeneration and impaired neuromuscular function [3–8]. In most cases, disease progression is severe enough to result in early death. Despite significant advances in clinical studies, experimental evidence of the pathogenicity of *ELAC2* alleles or an indication of underlying mechanisms leading to neuronal dysfunction is still missing.

RNase Z is an enzyme with homologs widely distributed among eukaryotes, archaea, and bacteria. It comes in two forms: RNase Z^S^, a short form found in all three domains of life; and RNase Z^L^, a Eukarya-specific long form [9]. The relative distribution of the corresponding genes varies among eukaryotes. Budding yeast, nematodes, and fruit flies carry only the *RNase Z*^*L*^ form. Humans, on the other hand, have both forms – *ELAC1* encodes RNase Z^S^ and *ELAC2* encodes RNase Z^L^. Both paralogs possess the same endonucleolytic cutting activity [10,11], but their intracellular distribution is distinct, and their roles in tRNA metabolism appear to be nonredundant [12,13]. RNase Z^S^ localizes primarily in the cytosol, where it recycles damaged tRNAs [14]. RNase Z^L^ is found in the nucleus and mitochondria, where it processes the 3’-ends of pre-tRNA encoded in the DNA of respective organelles. The RNase Z^L^ paralog contains both nuclear localization signal (NLS) and a mitochondrial targeting sequence (MTS). Its presence within two subcellular compartments is ensured by alternative translation initiation. *Drosophila* RNase Z has its MTS sandwiched between two methionine residues [15]. When translation starts from the first one, the MTS peptide targets the protein to mitochondria; when translation begins from methionine at position 24, the NLS translocates the protein to the nucleus [16].

Eukaryotic RNase Z is a ubiquitous protein, and its presence has been reported in multiple cell types in different tissues [17]. In *Drosophila*, as in many other species, it plays a vital role – the knockout (KO) alleles of the *RNase Z* gene cause early larval lethality [18]. Accumulating evidence shows that RNase Z has several molecular functions. Among identified targets for endonucleolytic cleavage are polycistronic mitochondrial transcripts, nuclear-encoded tRNA, microRNA, and small nucleolar RNA [15,19–22]. Correspondingly, RNase Z is involved in many processes, including cell growth and proliferation, cell cycle, regulation of gene expression, and mitochondrial stress response [16,18,23–27]. Given the ubiquitous presence and numerous activities of RNase Z, it is unsurprising that hypomorphic alleles of the gene lead to variable phenotypes and pleiotropic effects. The knockdown of *Drosophila RNase Z* results in diverse outcomes depending on the cell type in which RNA interference is initiated [28]. In general, mitotically replicating cells and cells with high metabolic demands are more sensitive to reduced RNase Z activity. In humans, hypomorphic variants of RNase Z^L^ are reportedly connected to the early development of prostate cancer [29,30], cardiomyopathy [2], and neurological disease [8]. Given that the fruit fly can mirror many human disorders, including those of the nervous system, we employed *Drosophila* genetics to establish the causal contribution of mutant RNase Z variants to neuropathology.

In this report, we present CRISPR/Cas9-generated fly models wherein *RNase Z* is mutated in a neuron-specific manner. Mutant flies undergo neurodegeneration, leading to motor and learning defects. Notably, restricting the presence of the mutant form of RNase Z to neuronal mitochondria was sufficient to produce neurological phenotypes. Altogether, our data not only provide a direct connection between RNase Z and neuropathology but, more importantly, identify mutant mitochondrial RNase Z as a central contributor to disease.

## Results

### Neurodegeneration in RNase Z mutants

Mutations in the *RNase Z* gene produce pleiotropic phenotypes in humans and flies, leading to multi-system dysfunction [2,8,18]. To assess the functional requirement of RNase Z in the nervous system, we employed CRISPR-TRiM [31]. Previously, we showed that this approach is specific and highly efficient at targeting endogenous RNase Z [32]. To achieve a tissue-specific knockout of the *RNase Z* gene (*Elav-Cas9 > RNZ*^*KO*^) we expressed Cas9 under the pan-neuronal *elav* promoter (Table 1) These flies developed into adulthood but died within 24–48 hrs after eclosion. The adults displayed strong motor impairments, with flies unable to stand, walk, jump, or fly. Instead, they remained supine with minor leg or wing twitching. The observed lethality and locomotor phenotypes suggest that neuronal RNase Z is indispensable for fly viability and motor activity.

**Table 1:** Experimental models.

Complete genotype	Abbreviation ^a^	Description
elav-Cas9/U6-3xgRNA^Z^	*Elav-Cas9 > RNZ* ^*KO*^	Neuronal knockout of endogenous RNase Z
elav-Cas9/U6-3xgRNA^Z^; + /gZ^ + ^-PAM^∆^	*Elav-Cas9 > RNZ* ^*+*^	Neuronal knockout of RNase Z rescued with Cas9-resistant wild type RNase Z encoded by the gZ^ + ^-PAM^∆^ transgene
elav-Cas9/U6-3xgRNA^Z^; + /gZ^T494I^-PAM^∆^	*Elav-Cas9 > RNZ* ^*T494I*^	Neuronal knockout of RNase Z rescued with Cas9-resistant RNase Z^T494I^ variant
elav-Cas9/U6-3xgRNA^Z^; + /gZ^∆MTS^-PAM^∆^	*Elav-Cas9 > mt-RNZ* ^*KO*^	Neuronal knockout of RNase Z rescued with Cas9-resistant RNase Z variant without MTS
elav-Cas9/U6-3xgRNA^Z^; gZ^∆MTS^-PAM^∆^/gZ^T494I^-PAM^∆^	*Elav-Cas9 > mt-RNZ* ^*T494I*^	Neuronal knockout of RNase Z rescued with two Cas9-resistant forms of RNase Z, one without MTS, and the other encoding Thr494Ile variant

^a^*Elav-Cas9* indicates the name of a neuron-specific promoter *elav* that drives a tissue-specific expression of the Cas9 enzyme; symbol > indicates that Cas9 to its left is targeting the *RNase Z* (*RNZ*) gene to its right; uppercase indexes – KO, + , T494I – indicate the result of the combined action of Cas9 and rescue: *RNZ*^*KO*^ – absence of RNase Z; *RNZ*^*+*^ – presence of wild-type RNase Z; *RNZ*^*T494I*^ – presence of mutant RNase Z.

**Fig 1:**
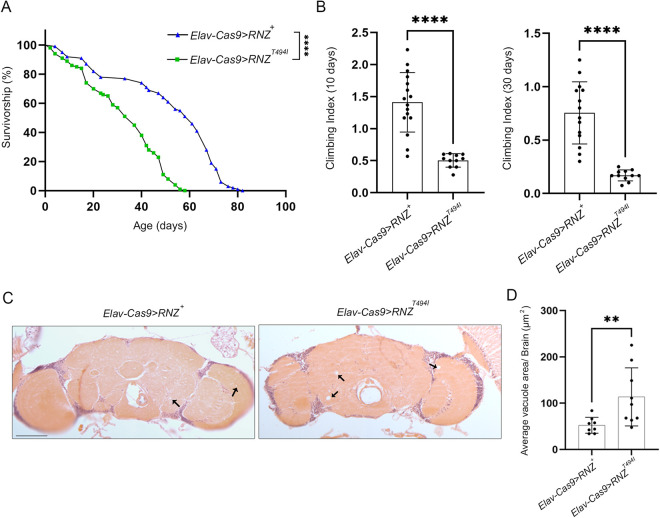
Neuronal RNase Z KO rescued with wild type (*Elav-Cas9 > RNZ*^*+*^) and mutant (*Elav-Cas9 > RNZ*^*T494I*^) copy of RNase Z. In all experimental models, the wild-type and disease-associated protein variants are supplied in amounts defined by the activity of the natural *RNase Z* promoter. (A) Survival rates for control *Elav-Cas9 > RNZ*^*+*^ and mutant *Elav-Cas9 > RNZ*^*T494I*^ flies (n = 100 for each genotype). ****P < 0.0001 (Mantel-Cox test). (B) Negative geotaxis expressed as a climbing index for control and mutant flies at 10 and 30 days of age (n > 100 for each genotype). ****p < 0.0001 (unpaired Student’s t-test). (C) Histological sections of the fly brain showing vacuolization in control and mutant adults on day 30 after eclosion. The arrows point to the vacuoles. Scale bar represents 100µm. (D) Quantification of vacuoles in the serial histological sections of *Elav-Cas9 > RNZ*^*+*^ (n = 8) and *Elav-Cas9 > RNZ*^*T494I*^ (n = 9) flies. **p < 0.0021 (unpaired Student’s t-test). Error bars indicate mean ± SD.

The Thr520Ile missense substitution was among the first few alleles of human *ELAC2/RNase Z*^*L*^ linked to a broad spectrum of neurological manifestations [2,7]. To validate the pathogenicity of the Thr520Ile substitution, we generated a fly line that carries an equivalent mutant variant Thr494Ile in all neurons (*Elav-Cas9 > RNZ*^*T494I*^) (Table 1). These flies exhibited normal development and produced viable adults. However, they showed reduced longevity (Fig 1A) and a progressive loss in locomotor ability (Fig 1B). Given that these phenotypes could be a sign of a neurological condition, we examined histological brain slices for vacuolization, a well-established proxy for neurodegeneration. In mutant flies, we observed more and larger vacuoles, with an average vacuole area of 113.6 µm^2^ compared to 52.3 µm^2^ in age-matched controls (Fig 1C and 1D). This analysis supports our suggestion that the *Elav-Cas9 > RNZ*^*T494I*^ flies have a progressive neurodegenerative phenotype.

### Impaired neuromuscular communication in RNase Z mutants

**Fig 2:**
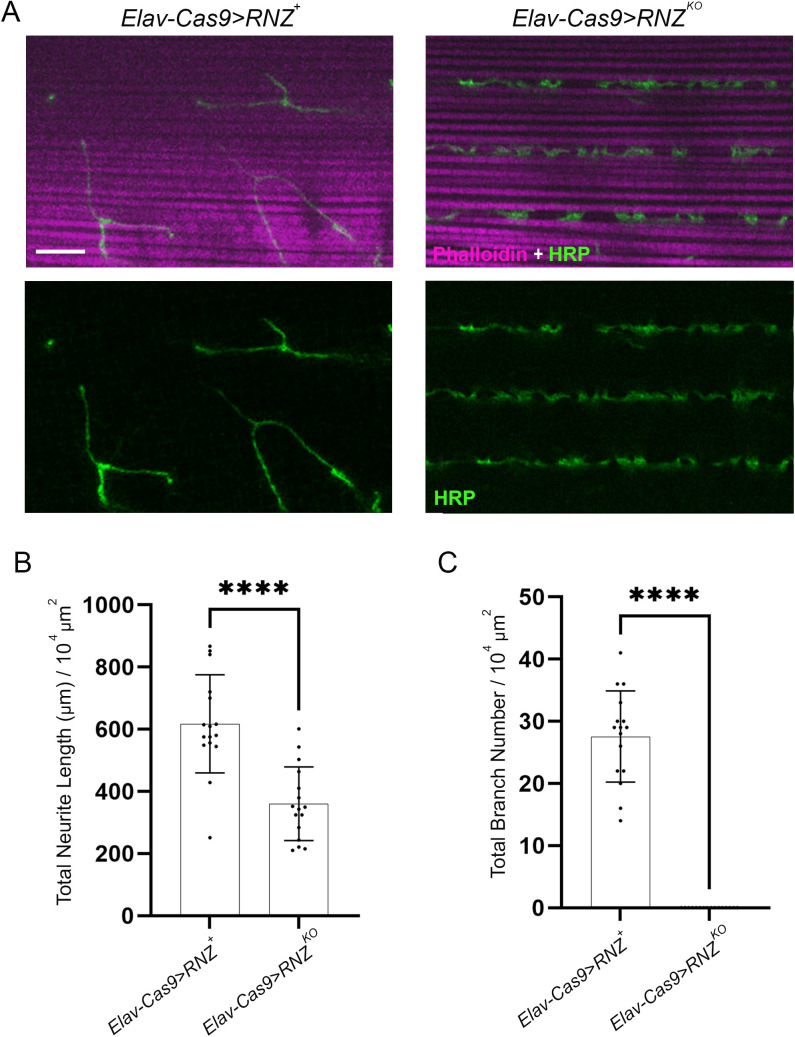
Morphology of NMJ synapses at DLM of flies deprived of neuronal RNase Z. (A) DLM NMJ synapses in 1-day-old control (*Elav-Cas9 > RNZ*^*+*^) and mutant (*Elav-Cas9 > RNZ*^*KO*^) flies were visualized via staining of motoneurons with FITC-conjugated HRP (green) and muscle fibers with Phalloidin (magenta). The scale bar represents 20 µm. Total neurite length (B) and branch number (C) per muscle area were quantified (n = 15-16). ****p < 0.0001 (unpaired Student’s t-test). Error bars indicate the mean ± SD.

Given that flies, deprived of neuronal RNase Z and rescued with the Thr494Ile variant, displayed signs of neurodegeneration, we next assessed innervation of the dorsal longitudinal muscle (DLM) critical for insect flight ability. The synaptic connectivity at the neuromuscular junction (NMJ) was quantified by measuring neurite length and branch number. In control animals (Fig 2), we found an innervation pattern characterized by extensive branching (26–27 per 10^4^ μm^2^) with a total neurite length of 613 μm (per 10^4^ μm^2^), a morphology conducive to proper synaptic transmission. On the contrary, in *Elav-Cas9 > RNZ*^*KO*^ flies, we found gross morphological abnormalities. Compared to the wild-type control, the neurites of motor neurons in the *Elav-Cas9 > RNZ*^*KO*^ flies were shorter (360 μm per 10^4^ μm^2^) and ran parallel to the long axis of the muscle fibers, lacking any branching (Fig 2). These data show a significant reduction of neuromuscular contacts in flies without neuronal RNase Z, which is consistent with their locomotor impairment.

**Fig 3:**
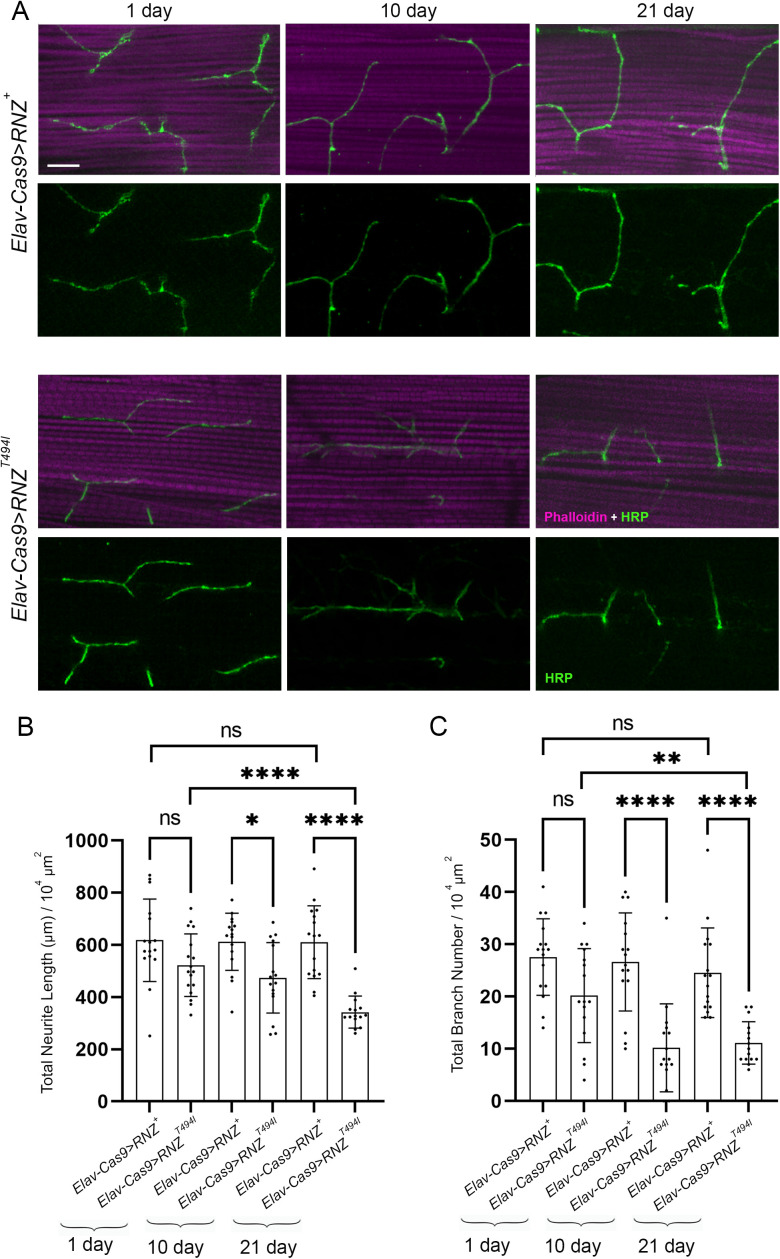
Age-dependent synaptic denervation of DLM in flies deprived of neuronal RNase Z and rescued with the RNase Z^T494I^ variant. (A) DLM NMJ synapses in 1-, 10-, and 21-day-old control (*Elav-Cas9 > RNZ*^*+*^) and mutant (*Elav-Cas9 > RNZ*^*T494I*^) flies stained as in Fig 2. The scale bar represents 20 µm. Total neurite length (B) and branch number (C) per muscle area were quantified (n = 15-16). ns., no statistical significance; *p < 0.0332, **p < 0.0021, ***p < 0.0002 and ****p < 0.0001 (unpaired Student’s t-test). Error bars indicate the mean ± SD.

Next, we looked at flies, in which neuron-specific KO of RNase Z is rescued with the Thr494Ile variant of RNase Z. The median of their lifespan is 35 days (Fig 1), which allowed us to study the structural integrity of NMJs at several time points (Fig 3A). On day 1 after eclosion, control *Elav-Cas9 > RNZ*^*+*^ and mutant *Elav-Cas9 > RNZ*^*T494I*^ flies showed little to no difference in total neurite length and branch number (Fig 3B and 3C). On days 10 and 21, while the innervation pattern did not change in control flies, mutants showed progressive deterioration of NMJ synapses (Fig 3B and 3C). In a span of three weeks after eclosion, the total neurite length reduced from an average of 522–343 μm, and the branch number decreased from 20 to 11. Thus, functional RNase Z is required in neurons to support the synaptic structure.

**Fig 4:**
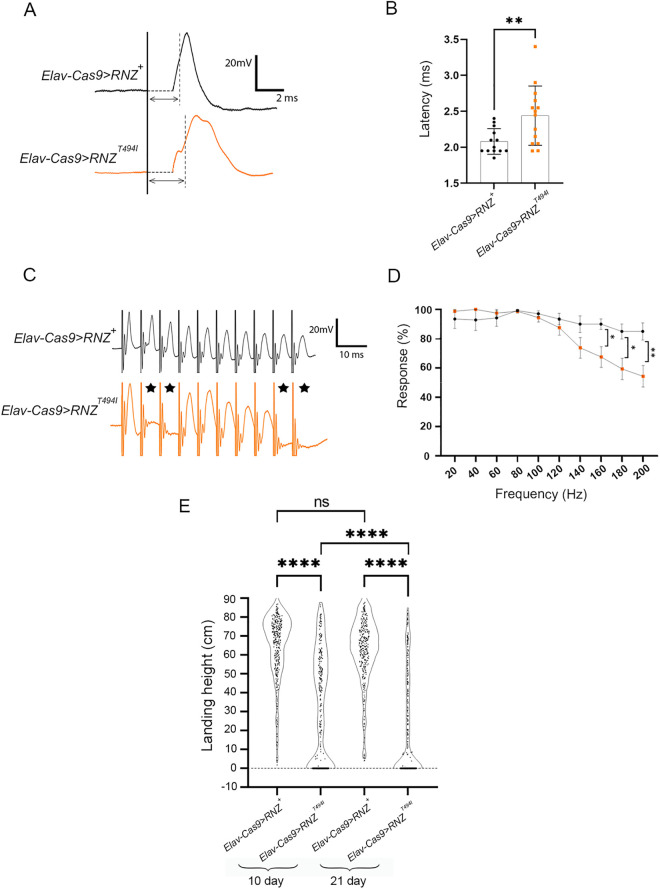
Neuromuscular communication and flight performance in flies deprived of neuronal RNase Z and rescued with the RNase Z^T494I^ variant. (A) Representative trace files showing a latency between the stimulus (marked by solid vertical line) applied to the GF and the DLM depolarization (at its half-maximal amplitude – dashed line) in flies of indicated genotypes. The double-headed arrows indicate the latency period. (B) The transmission latency was quantified based on multiple independent repeats for control *Elav-Cas9 > RNZ*^*+*^ (n = 13) and mutant *Elav-Cas9 > RNZ*^*T494I*^ (n = 14) flies. (C) Representative trace files showing spikes at DLM in response to a train of high-frequency stimulation. Note that in contrast to control flies, the *Elav-Cas9 > RNZ*^*T494I*^ mutants are unable to follow each stimulus with a corresponding spike of depolarization (marked with black stars). (D) Stimulus trains were delivered at progressively higher frequencies (20 – 200 Hz), and the response rate was recorded for *Elav-Cas9 > RNZ*^*+*^ (n = 15) and mutant *Elav-Cas9 > mt-RNZ*^*T494I*^ (n = 15) flies. *p < 0.0332, **p < 0.0021 (Mann-Whitney non-parametric t-test). (E) Data from the flight performance assay displayed in violin plots for control (*Elav-Cas9 > RNZ*^*+*^) and mutant (*Elav-Cas9 > RNZ*^*T494I*^) flies. Each dot represents a single fly. Sample size and average landing height for each plot: *Elav-Cas9 > RNZ*^*+*^ day 10 (n = 336) landing average 65.0 cm; *Elav-Cas9 > RNZ*^*T494I*^ day 10 (n = 296) landing average 34.1 cm; *Elav-Cas9 > RNZ*^*+*^ day 21 (n = 313) landing average 62.8 cm; *Elav-Cas9 > RNZ*^*T494I*^ day 21 (n = 369) landing average 21.0 cm. ns., not significant and ****p-value <0.0001 (unpaired Student’s t-test).

To assess a potential disruption in neuromuscular communication, we next measured electrophysiological activity across NMJs. As a model, we used the Giant Fiber (GF) pathway, a well-characterized neuronal circuit that, when stimulated, brings the signal to the DLM and thus triggers a flight behavior. Control and mutant flies were tested on day 21 after eclosion, a time point when the difference in DLM innervation is easily perceptible (Fig 3). Following a previously described protocol [33], we activated GF with an electrical stimulation into the fly head and recorded the action potential from the flight muscle (Fig 4A). As expected, in control flies, the DLM response to a single stimulus came with a stereotypic latency of 2.0 ms. In mutants, the latency period increased up to 2.4 ms (Fig 4B). We also examined the ability of the GF pathway to faithfully transmit high-frequency stimulation [34]. Control and mutant flies received 10 sets of 10 stimuli at progressively higher frequencies (20 – 200 Hz). We found that at stimulation frequencies higher than 120 Hz, mutant flies, *Elav-Cas9 > RNZ*^*T494I*^, displayed a reduced ability to follow stimulation compared to control *Elav-Cas9 > RNZ*^*+*^ flies (Fig 4C and 4D).

A loss of structural integrity and decreased signal transmission at NMJs suggests that flight behavior could also be affected in mutant flies. To test this, we analyzed the flight ability in mutant and control animals on days 10 and 21 after eclosion, using a flight performance test (S1 Fig). Briefly, this test involves dropping flies into a cylinder, followed by recording the landing position to evaluate how effectively the flies initiate flight upon release. *Elav-Cas9 > RNZ*^*+*^ flies consistently performed well, showing average landing heights of 65 and 63 cm at two respective time points. On the contrary, the *Elav-Cas9 > RNZ*^*T494I*^ mutants performed poorly across both days, with average landing heights of 34 cm (day 10) and 21 cm (day 21) (Fig 4E).

Taken together, our data show that flies lacking functional RNase Z in neurons experience a reduction of DLM innervation, leading to the inability to properly transmit signals at NMJs. Consequently, these flies exhibit locomotor deficit that rapidly declines with age.

### Impaired associative learning in RNase Z mutants

Patients carrying mutant ELAC2/RNase Z^L^ variants may display microcephaly and cognitive defects [2–4]. Initial inspection of the *Elav-Cas9 > RNZ*^*KO*^ animals showed that their brains were visibly smaller (S2 Fig). Further analysis of the adult brain anatomy using the nc82 antibody revealed that a paired neuropil structure above the antennal lobes was absent (S3 Fig). This structure was determined to be the mushroom body (MB), the learning and memory center in the *Drosophila* brain. The MB comprises thousands of intrinsic neurons, Kenyon cells (KC), that form two clusters in the back of the brain. KC axons bundle together and extend toward the anterior region, where they split and extend further vertically in the dorsal direction and horizontally in the medial direction. The vertical projections form α and α’ lobes, and the horizontal projections are β, β’, and γ lobes.

**Fig 5:**
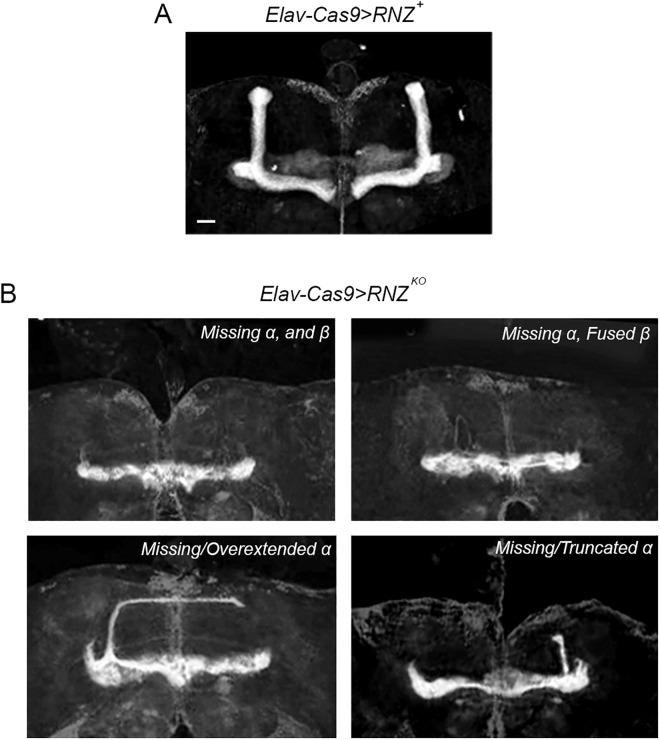
Mushroom body morphology in flies deprived of neuronal RNase Z. Representative images of MB of (A) *Elav-Cas9 > RNZ*^*+*^ control and (B) Elav*-Cas9 > RNZ*^*KO*^ mutant flies stained with anti-FasII. Each panel in (B) shows a distinct phenotype observed in flies devoid of neuronal RNase Z: missing, overextended, truncated, or fused α/α’ and β/β’ lobes. The scale bar represents 20 µm.

For a clearer view of the MB and a better evaluation of the structural damage produced by mutant *RNase Z* alleles, we stained adult brains with the Fasciclin II antibody (FasII). The FasII protein is particularly enriched in the KC axonal projections of α/α’ and β/β’ lobes. In *Elav-Cas9 > RNZ*^*KO*^ flies, the anatomy of the MB was severely deformed. Fig 5 shows examples of the damage, where α/α’ and β/β’ lobes were either missing, truncated, fused, or overextended. To determine when this damage first arises, we examined the MB structure at two pre-adult developmental time points – larva and pharate adult. We found that the structural abnormalities emerge during the pharate adult stage and become more prominent in newly eclosed adults (S4 Fig).

**Fig 6:**
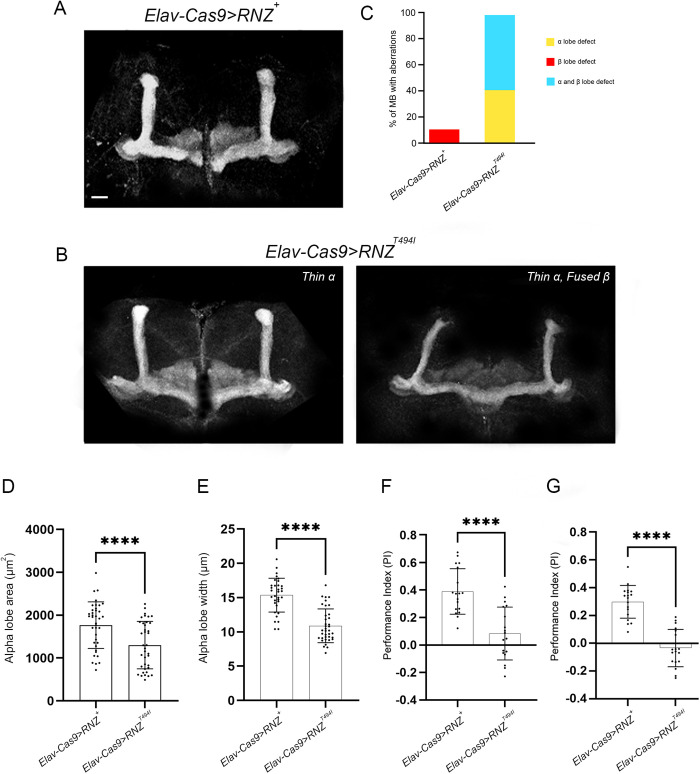
Mushroom body morphology and learning ability in flies deprived of neuronal RNase Z and rescued with the RNase Z^T494I^ variant. Representative images of MB of (A) *Elav-Cas9 > RNZ*^*+*^ control and (B) *Elav-Cas9 > RNZ*^*T494I*^ mutant flies stained with anti-FasII antibodies. The scale bar represents 20 µm. (C) Quantification of MBs with α/β lobe defects. Aberrant morphology of the α/α’ lobes was assessed by measuring lobe area (D) and diameter (E). Each dot represents the average value of the corresponding parameters from two α/α’ lobes of a single brain. At least 35 brains per genotype were studied. ****p < 0.0001 (unpaired Student’s t-test). Error bars indicate the mean ± SD. (F, G) Learning and memory was assessed via olfactory conditioning assay. For the long-term memory (F), flies were trained using the appetitive conditioning protocol and tested 24 hrs later. For the short-term memory (G), flies were trained using the aversive conditioning protocol and tested within 1 hr after that. The PI is calculated for each set of ~50 flies with the total of 10-12 sets being tested (n > 500). ****p < 0.0001 (unpaired Student’s t-test). Error bars indicate the mean ± SD.

In adult *Elav-Cas9 > RNZ*^*T494I*^ flies carrying a mutant variant of RNase Z in neurons, the structural damage to MB was less severe, though the morphological differences between mutant and control animals were clear and quantifiable (Fig 6A-E). Using two parameters – lobe area and width, we established that the α/α’ lobes were significantly thinner. In addition, 56% of MBs from mutant animals possessed fused β/β’ lobes. Collectively, our data show that the structural integrity of the MB depends on the presence of functional RNase Z in neurons.

Given the abnormal morphology of the MB, a structure essential for olfactory learning and memory, we next assessed these processes in our mutants (S5 Fig). First, control and mutant flies were trained to associate a particular odor with a reward (appetitive conditioning) or punishment (aversive conditioning). The appetitive conditioning with the sugar reward was used to assay long-term memory (LTM), and aversive conditioning with electric shock punishment to assay short-term memory (STM). We used 4-methylcyclohexanol (MCH) and 3-octanol (OCT) as odors, for neither of which flies have an inherent preference in the absence of training (S6 Fig). After training, flies were tested using a T-maze by presenting both odors simultaneously in the absence of a reward or punishment. The control flies, *Elav-Cas9 > RNZ*^*+*^, showed a robust performance of LTM and STM, as they strongly favored the odor associated with the reward and avoided the one associated with punishment (Fig 6F and 6G). In contrast, *Elav-Cas9 > RNZ*^*T494I*^ mutants displayed a pronounced deficit of LTM and STM, as their performance indices on average were close to 0, similar to those of untrained flies. These results are consistent with the abnormal MB structure in mutants and indicate that neuronal RNase Z is required for functional olfactory learning circuitry in the *Drosophila* brain.

### Loss of neuronal RNase Z confers functional damage to mitochondria

Multiple clinical studies have reported reduced respiratory complex I (CI) activity in patients harboring pathogenic ELAC2/RNase Z^L^ variants, signifying mitochondrial dysfunction [2,7]. Here, we studied CI activity in control (*Z*^*24*^;*RNZ*^*+*^) and mutant (*Z*^*24*^;*RNZ*^*T494I*^) flies homozygous for the *Z*^*24*^ knockout allele and rescued with *RNZ*^*+*^ and *RNZ*^*T494I*^ transgenes, respectively [18,35]. As a positive control for CI inactivation, we included flies, in which RNase Z was knocked down via RNA-interference (*Mef2 > RNZi*). In-gel assay with NADH substrate revealed a 63% reduction of CI activity in *Z*^*24*^;*RNZ*^*T494I*^ and 89% reduction in *Mef2 > RNZi* flies compared to *Z*^*24*^;*RNZ*^*+*^ control (S7 Fig). This experiment demonstrates that, just as in humans, the Thr494Ile variant of RNase Z impairs CI activity in flies.

**Fig 7:**
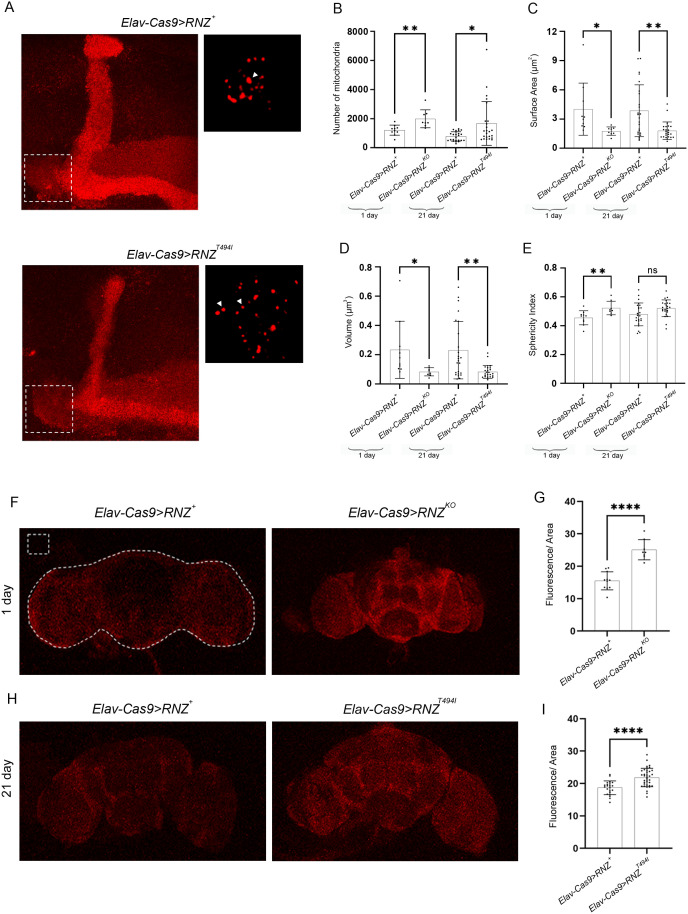
Mitochondrial morphology and ROS levels in flies deprived of neuronal RNase Z and rescued with the RNase Z^T494I^ variant. (A) Mitochondria of the MB were visualized with the neuron-specific reporter *mitoDsRed* (*Elav-Gal4;UAS-mitoDsRed*). The panels on the left are MIPs, a maximum intensity projection of all the slices imaged, and the dotted box denotes the 25x25 μm2 Region of Interest (ROI) chosen for analysis. The panels on the right are high resolution MIPs of three consecutive slices, a small representative subset of mitochondria from ROI shown on the left. The arrowheads point to representative mitochondria. (B-E) Quantification of (B) mitochondrial number, (C) surface area (μm2), (D) volume (μm3), and (E) sphericity index, in 1-day-old flies *Elav-Cas9 > RNZ*^*+*^ (n = 10), *Elav-Cas9 > RNZ*^*KO*^ (n = 8), and 21-day-old flies *Elav-Cas9 > RNZ*^*+*^ (n = 24), *Elav-Cas9 > RNZ*^*T494I*^ (n = 24). Each plot point represents the average of the measured values from a single brain. ns., not significant, *p-value<0.0332, **p-value<0.0021 (unpaired student’s t-test). Error bars indicate the mean ± SD. (F, H) Representative images of DHE staining of 1 and 21-day-old brains of *Elav-Cas9 > RNZ*^*+*^, *Elav-Cas9 > RNZ*^*KO*^, and *Elav-Cas9 > RNZ*^*T494I*^ flies. The dotted line around the brain denotes the area chosen for integrated density measurement, and the dotted box denotes the region used for background fluorescence measurement. (G, I) Quantification of DHE fluorescence. Average fluorescence/area and sample size for plots in (G) *Elav-Cas9 > RNZ*^*+*^ 15.5 (n = 11); *Elav-Cas9 > RNZ*^*KO*^ 25.1 (n = 8), and in (I) *Elav-Cas9 > RNZ*^*+*^ 18.7 (n = 22); *Elav-Cas9 > RNZ*^*T494I*^ 21.3 (n = 37). ****p < 0.0001 (unpaired student’s t-test). Error bars indicate the mean ± SD.

Next, to determine if brain mitochondria are affected in flies lacking neuronal RNase Z and rescued with the Thr494Ile variant, we examined organelle morphology in adult flies with the mito-DsRed transgene as a reporter to visualize mitochondria. We looked at mitochondria in the MB neurons in the area close to the bifurcation point of the α and β lobes (Fig 7A). As we compared *Elav-Cas9* > *RNZ*^*KO*^ flies to the age-matched controls, we found that mitochondria in neurons lacking RNase Z were fragmented. Similarly, mitochondria of *Elav-Cas9 > RNZ*^*T494I*^ flies, as illustrated in the high-resolution image (Fig 7A, right panel), displayed parameters characteristic of fragmentation, as they measured more numerous with smaller surface area and volume (Fig 7B-E). This analysis shows that removing wild-type RNase Z from neurons or replacing it with the mutant form impacts mitochondrial number, size, and shape.

Assuming that mitochondrial fragmentation is indicative of functional damage, our data would be consistent with previously published studies in humans, mice, and flies, all showing that loss of ELAC2/RNase Z^L^ impairs mitochondrial transcript processing, lessens assembly of ETC complexes, and causes a profound mitochondrial dysfunction [2,15,22]. Importantly, our prior work established that flies with systemic mt-RNase Z KO accumulate higher ROS levels because of disabled ETC [16]. To confirm that organelle fragmentation found in our mutants is indeed a manifestation of mitochondrial dysfunction, we next assessed ROS levels in brains of flies lacking neuronal RNase Z and rescued with the Thr494Ile variant. Using dihydroethidium (DHE) dye as an indicator, we found that *Elav-Cas9 > RNZ*^*K*O^ flies displayed a 62% increase in ROS content compared to the age-matched controls (Fig 7F and 7G). Similarly, ROS levels were elevated by 17% in brain neurons of *Elav-Cas9 > RNZ*^*T494I*^ flies (Fig 7H and 7I). Altogether, our data show that neuron-specific KO and rescue with mutant RNase Z variant damages the structural and functional integrity of mitochondria.

### Mitochondrial RNase Z is necessary for locomotor and learning ability

Clinical studies have associated mutant alleles of *ELAC2/RNase Z*^*L*^ with mitochondrial disease. However, the organelle-specific contributions to the development of pathological symptoms have not yet been assessed. To address this issue, we separated intramitochondrial RNase Z activity from its other functions in neuronal cells by generating *Elav-Cas9 > mt-RNZ*^*KO*^ flies. These animals have a neuron-specific KO of RNase Z, and they carry a rescue transgene encoding the wild-type protein without the MTS (S8 Fig). Therefore, these flies do not have mt-RNase Z in their neurons (Table 1). They progressed through all stages of development and became viable adults but died within 1–2 days of eclosion.

**Fig 8:**
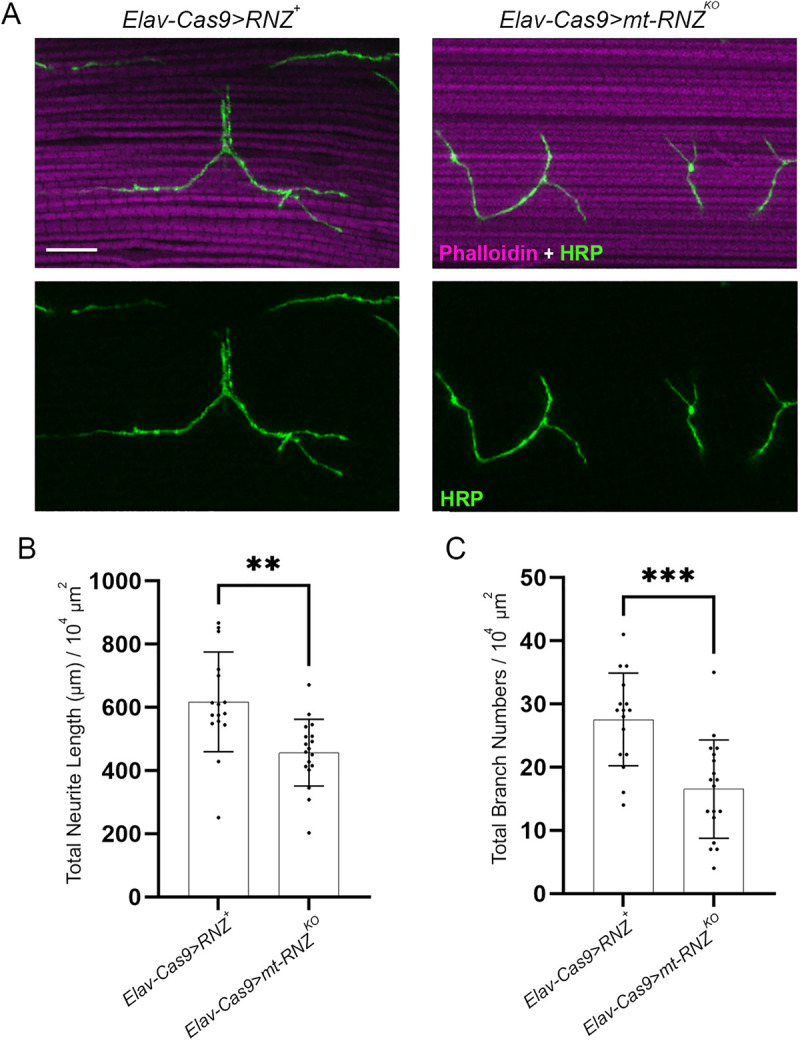
Morphology of NMJ synapses at DLM of flies deprived of neuronal mt-RNase Z. (A) DLM NMJ synapses in 1-day-old control (*Elav-Cas9 > RNZ*^*+*^) and mutant (*Elav-Cas9 > mt-RNZ*^*KO*^) flies stained as in Fig 2. The scale bar represents 20 µm. Total neurite length (B) and branch number (C) per muscle area were quantified (n = 15-16). **p < 0.0021 and ***p < 0.0002 (unpaired Student’s t-test).

**Fig 9:**
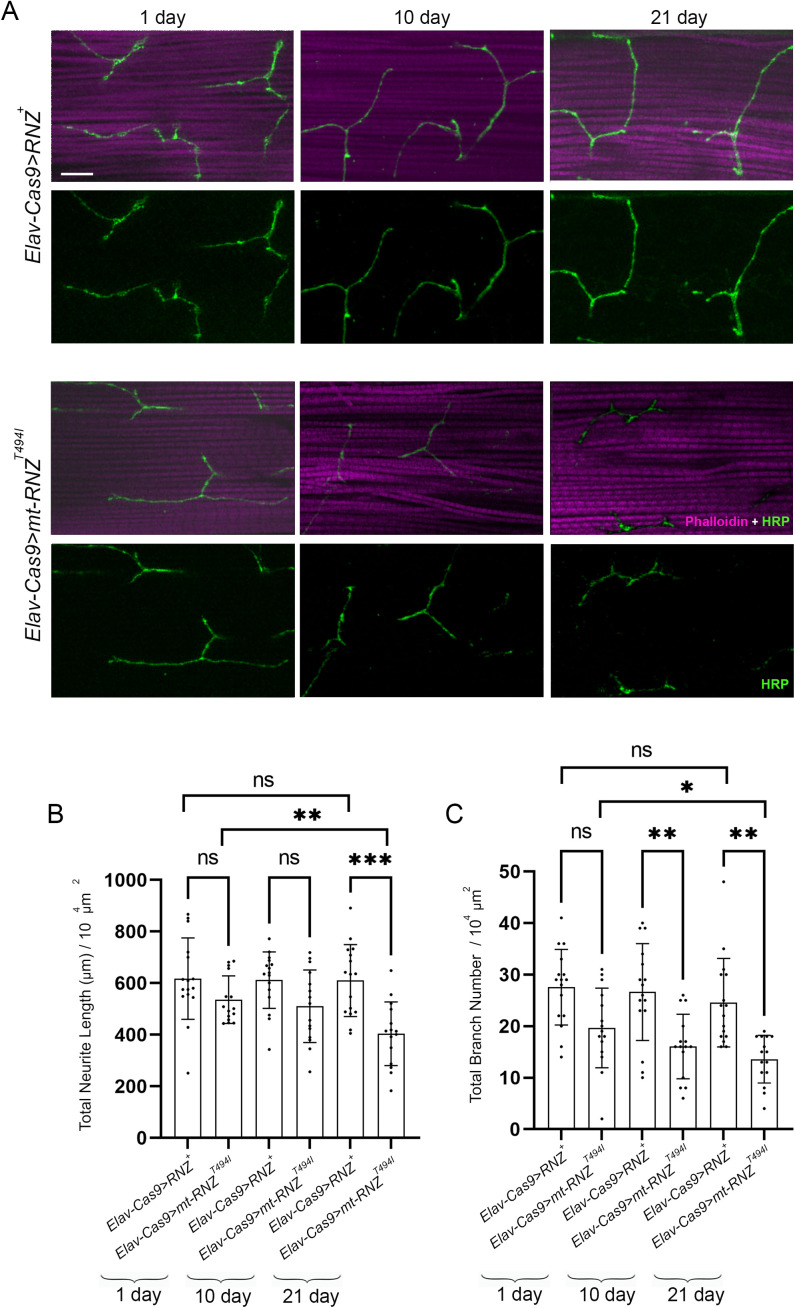
Age-dependent synaptic denervation of DLM in flies deprived of neuronal mt-RNase Z and rescued with the RNase Z^T494I^ variant. (A) DLM NMJ synapses in 1-, 10-, and 21-day-old control (*Elav-Cas9 > RNZ*^*+*^) and mutant (*Elav-Cas9 > mt-RNZ*^*T494I*^) flies stained as in Fig 2. The scale bar represents 20 µm. Total neurite length (B) and branch number (C) per muscle area were quantified (n = 15-16). ns., no statistical significance, *p < 0.0332, **p < 0.0021, ***p < 0.0002, and ****p < 0.0001 (unpaired student’s t-test). Error bars indicate the mean ± SD.

**Fig 10:**
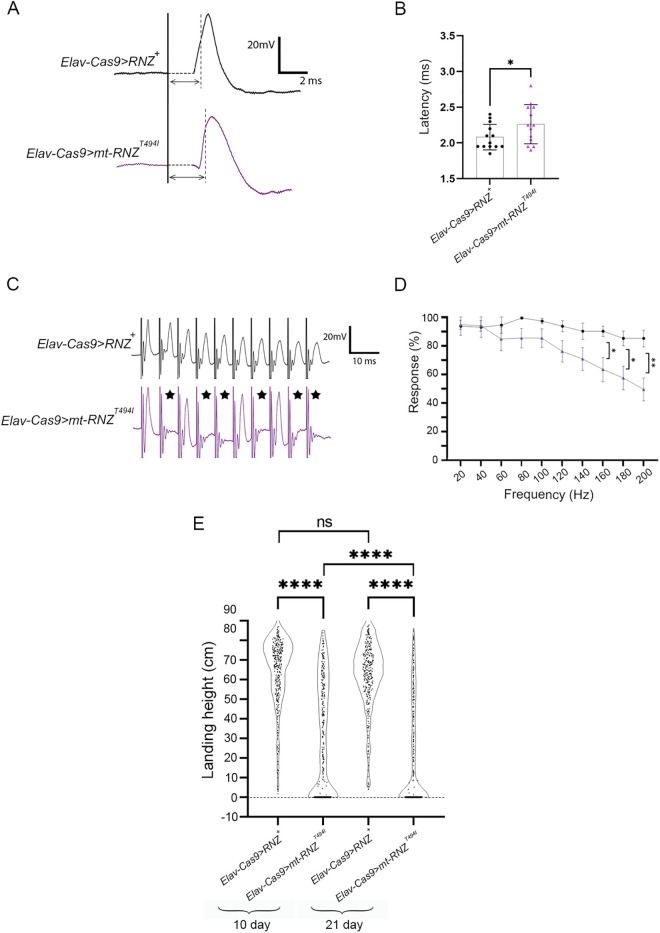
Neuromuscular communication and flight performance in flies deprived of neuronal mt-RNase Z and rescued with the RNase Z^T494I^ variant. (A) Representative trace files showing a latency (double-headed arrows) between the stimulus (marked by solid vertical line) applied to the GF and the DLM depolarization (at its half-maximal amplitude – dashed vertical line) in flies of indicated genotypes. (B) The transmission latency was quantified based on multiple independent repeats for control *Elav-Cas9 > RNZ*^*+*^ (n = 13) and mutant *Elav-Cas9 > mt-RNZ*^*T494I*^ (n = 13) flies. (C) Representative trace files showing spikes at DLM in response to a train of high-frequency stimulation. Note that in contrast to control flies, the *Elav-Cas9 > mt-RNZ*^*T494I*^ mutants are unable to follow each stimulus with a corresponding spike of depolarization (marked with black stars). (D) Stimulus trains were delivered at progressively higher frequencies (20 – 200 Hz), and the response rate was recorded for *Elav-Cas9 > RNZ*^*+*^ (n = 15) and mutant *Elav-Cas9 > mt-RNZ*^*T494I*^ (n = 14) flies. *p < 0.0332, **p < 0.0021 (Mann-Whitney non-parametric t-test). (E) Data from the flight performance assay displayed in violin plots for control (*Elav-Cas9 > RNZ*^*+*^) and mutant (*Elav-Cas9 > mt-RNZ*^*T494I*^) flies. Each dot represents a single fly. Sample size and average landing height for each plot: *Elav-Cas9 > RNZ*^*+*^ day 10 (n = 336) landing average 65.0 cm; *Elav-Cas9 > mt-RNZ*^*T494I*^ day 10 (n = 325) landing average 31.3 cm; *Elav-Cas9 > RNZ*^*+*^ day 21 (n = 313) landing average 62.8 cm; *Elav-Cas9 > mt-RNZ*^*T494I*^ day 21 (n = 332) landing average 21.0 cm. ns., no statistical significance and ****p-value <0.0001 (unpaired Student’s t-test).

To determine if mt-RNZ^T494I^ has neuropathological potential, we generated a mutant fly line, *Elav-Cas9 > mt-RNZ*^*T494I*^, in which neuron-specific KO of mt-RNase Z is rescued with the Thr494Ile variant (Table 1). These mutant flies developed into viable adults but showed reduced longevity and a progressive decline in fitness (S9 Fig). Next, to gauge if mitochondrial RNase Z contributes to neuronal integrity and function, we studied motor and MB neurons in flies completely deprived of neuronal mt-RNase Z or rescued with the Thr494Ile variant. In *Elav-Cas9 > mt-RNZ*^*KO*^ flies, the motoneuron branching pattern showed a significant reduction in both the total neurite length (456.4 μm per 10^4^ μm^2^) and branch number (16 per 10^4^ μm^2^) (Fig 8A-C). *Elav-Cas9 > mt-RNZ*^*T494I*^ flies showed an age-dependent decay of NMJ synapses. The 21-day-old mutants had an average neurite length of 403.5 μm with 13 branches, while the age-matched control flies had a neurite length of 600 μm with 27 branches (Fig 9A-C). Consistent with the neuroanatomical disruption, our electrophysiological analyses revealed that the GF pathway in mutants responded to a single stimulus with an increased latency and was unable to follow high-frequency stimulation (Fig 10A-D). Furthermore, *Elav-Cas9 > mt-RNZ*^*T494I*^ flies displayed impaired flight behavior at two respective time points, with the average landing height markedly reduced from 53 cm in control to 31 cm in mutants (on day 10) and from 49 cm to 20 cm (on day 21) (Fig 10E).

**Fig 11:**
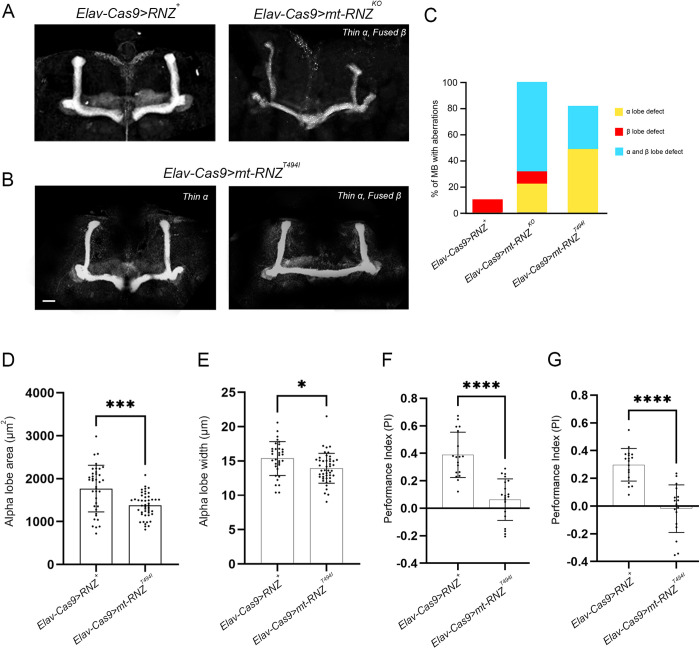
Morphology of MB neurons and cognitive function in flies deprived of neuronal mt-RNase Z and rescued with the RNase Z^T494I^ variant. Representative images of MB of (A) Elav*-Cas9 > RNZ*^*+*^ control and (B) *Elav-Cas9 > mt-RNZ*^*T494I*^ mutant flies stained with anti-FasII antibodies. The scale bar represents 20 µm. (C) Quantification of MBs with α/β lobe defects. Aberrant morphology of the α/α’ lobes was assessed by measuring lobe area (D) and diameter (E). Each dot represents the average value of the corresponding parameters from two α/α’ lobes of a single brain. At least 35 brains per genotype were studied. ****p < 0.0001 (unpaired Student’s t-test). Error bars indicate the mean ± SD. (F, G) Learning and memory was assessed via olfactory conditioning assay. For the long-term memory (F), flies were trained using the appetitive conditioning protocol and tested 24 hrs later. For the short-term memory (G), flies were trained using the aversive conditioning protocol and tested within 1 hr after that. The PI is calculated for each set of ~50 flies with the total of 10-12 sets being tested (n > 500). ****p < 0.0001 (unpaired Student’s t-test). Error bars indicate the mean ± SD.

Next, we examined the MB morphology and observed that flies deprived of neuronal mt-RNase Z displayed prominent abnormalities (Fig 11A and 11C). About 95% of *Elav-Cas9 > mt-RNZ*^*KO*^ flies had α/α’ lobes that were thin and overextended or thin and truncated, and 83% had fused β/β’ lobes. In *Elav-Cas9 > mt-RNZ*^*T494I*^ flies, the malformations of MB were less striking but still significant (Fig 11B and 11C). Based on our measurements of area and width, 96% of mutant flies had thinner α/α’ lobes, and 33% had their β/β’ lobes fused over the midline (Fig 11D and 11E). These flies also exhibited impaired learning/memory with performance indices averaging close to 0 for flies tested at either 2 or 24 hr after training (Fig 11F and 11G). Thus, this analysis shows that mitochondrial RNase Z is necessary for proper neuronal development and maintenance. Moreover, expression of mt-RNase Z^T494I^ in place of the wild-type protein in fly neurons is sufficient to induce neurological phenotypes.

## Discussion

### Targeting neuronal RNase Z causes a loss of locomotor function and shortened lifespan

*RNase Z* is ubiquitously expressed in different cell types. Humans harboring mutant alleles of this gene display multi-system defects, highlighting the pleiotropic consequences of RNase Z disruption. In the context of neurological disease, neuronal deficits can arise not only from neuron-intrinsic factors but also from non-neuronal cell contributions, including those from glia and muscle cells. To avoid confounding non-cell-autonomous effects and focus on neuron-intrinsic contributions to neuropathology, we employed the CRISPR-TRiM technique to generate flies with the KO of RNase Z in neurons (Table 1).

We found that flies devoid of neuronal RNase Z displayed severe motor defects and microcephaly. This extreme phenotype suggests a complete loss of neuronal control over locomotor activity as well as aberrant brain development, both characteristic of neuropathology. The severity of the phenotype arises from the twofold damage caused by the lack of RNase Z in the nucleus and mitochondria. Previously, we showed that loss of RNase Z prevents tRNA maturation, which limits the availability of cytoplasmic tRNA for translation and hampers protein synthesis [18]. The brain, in general, and neurons, in particular, are vulnerable to such proteostatic disturbances due to their reliance on protein synthesis for neurogenesis [36] and synaptic maintenance and transmission [37]. Furthermore, the presence of RNase Z in mitochondria is also indispensable. Without RNase Z, mitochondrial polycistronic transcripts are not processed, the respiratory complexes are not assembled, and energy production drops [16]. Cells with strict aerobic metabolism, such as neurons, are highly sensitive to energy limitations, and a disruption in energy dynamics alters the homeostatic mechanisms underlying neuronal network stability [38]. Therefore, nuclear and mitochondrial RNase Z may function synergistically to sustain neurodevelopment and neurological function.

Interestingly, neuronal RNase Z KO did not interfere with larval development or survivability. Animals developed through three instars, pupariated successfully, and underwent metamorphosis with no visible locomotor impairments. The apparent inconsistency between larval motility and adult locomotor deficiency originates from the timing of the Cas9-mediated disruption of *RNase Z* versus its zygotic expression. In our flies, production of Cas9 is driven by the *elav* promoter, which makes this enzyme available only at around 9–10 hours post-fertilization. Moreover, kinetic studies show that after Cas9 activation, it may take up to 10 hours before actual modification is introduced at the target site of the genome [39,40]. Notably, RNase Z zygotic expression starts within 2–3 hours after fertilization. Thus, it appears that the amount of RNase Z and tRNAs accumulated in proliferating neuroblasts and differentiating neurons during embryogenesis, prior to the knockout of genomic *RNase Z*, is sufficient to support successful preadult development. However, as this supply is depleted, the addition of new neurons, pruning, and regrowth of existing neurons during metamorphosis would be affected (S4 Fig), manifesting in microcephaly and loss of locomotion in adulthood. Thus, we conclude that neuronal RNase Z is required for nervous system remodeling during metamorphic transition from larva to adult, and mutant neuronal RNase Z gives rise to a range of neurodevelopmental phenotypes. At the same time, locomotor deficits, brain vacuolization, and neuronal morphological abnormalities also worsen with age; therefore, the pathology in flies encompasses both developmental and degenerative features.

Overall, our analysis of the *Elav-Cas9 > RNZ*^*KO*^ flies underscores the critical role of neuronal RNase Z in sustaining locomotor ability, ensuring proper neurodevelopment, and overall organismal viability.

### Neurological pathogenicity of the *RNase Z^T494I^* allele

To validate the pathogenicity of the Thr494Ile substitution, we combined the neuron-specific knockout and the rescue transgenic construct yielding pan-neuronal mutants *Elav-Cas9 > RNZ*^*T494I*^ (Table 1), which harbor the Thr494Ile form of RNase Z in neuronal cells only. The lifespan of these flies was far shorter (a median of 35 days) than that of control ones. They also showed a progressive decline of locomotor ability and vacuolization of brain, clear signs of neurodegeneration. Further analysis confirmed that the *Elav-Cas9 > RNZ*^*T494I*^ flies experience progressive deterioration of the glutamatergic motor neurons that innervate the flight muscle. Given that the function of these neurons is to relay signals from the CNS to the DLM flight muscle, synaptic denervation resulted in a decline in signal transmission, evidenced by electrophysiological tests. Correspondingly, mutant flies exhibited an age-dependent decline in flight ability.

In addition to motor defects, *RNase Z* mutation also disrupted associative olfactory learning, as *Elav-Cas9 > RNZ*^*T494I*^ flies displayed a pronounced deficit of short- and long-term memory. The formation, storage, and retrieval of *Drosophila* associative memory are dependent on the MB. Sensory information (*e.g.*, olfactory) is brought in through the projection neurons, then processed by KCs, and transmitted to the MB output neurons (MBON), which relay the signal to other areas of the brain and promote cue-driven behaviors [41]. The association of odors with an unconditioned stimulus (*e.g.*, electric shock, sugar reward) occurs within the MB lobes, where KC axons synapse on each other, on MBONs, and intersect with dopaminergic neurons [42–44]. Our inspection of the MB morphology in flies deprived of neuronal RNase Z and rescued with the RNase Z^T494I^ variant showed gross abnormalities in the structure of α/α’ and β/β’ lobes, indicating that the impairment of this circuitry could underlie the observed memory deficits.

The essential role of RNase Z in neurons can be attributed to its involvement in protein synthesis as a tRNA processing enzyme. The activity and maintenance of neurons rely on proteostasis, and studies in different model systems have shown that disruptions of the translation apparatus (*e.g.*, tRNA, initiation/elongation factors) or associated processes (*e.g.*, tRNA maturation, modification, and aminoacylation) could lead to neurodegeneration [45,46]. In *Drosophila*, loss of microRNA *miR-34* leads to a massive dysregulation of genes associated with translation, resulting in protein aggregates in the brain, which promote neurodegeneration [47]. Furthermore, human alanyl-tRNA synthetase (AARS) has been implicated in different disorders affecting the nervous system [48,49]. In mice, a missense mutation in the editing domain of AARS leads to mischarging of tRNA^Ala^, accumulation of misfolded proteins, and neurodegeneration [50]. Overall, progressive neuronal decline observed in the *Elav-Cas9 > RNZ*^*T494I*^ mutant is consistent with the emerging notion that dysregulation of protein translation could be a common mechanism for the failed neuronal maintenance.

Human patients carrying mutations of *ELAC2/RNase Z*^*L*^ experience a variety of neurological symptoms. The fly models we created and describe here recapitulate most of them, starting from reduced lifespan to brain lesions and behavioral changes. These observations suggest that the RNase Z protein has an evolutionarily conserved neuronal function. Collectively, our findings in flies corroborate the causal connection between the Thr520Ile mutation of human ELAC2/RNase Z^L^ and neurological symptoms in patients carrying this allele.

### Mitochondrial dysfunction and neurological phenotypes in *Elav-Cas9 > RNZ^T494I ^*flies

Elucidating the mechanisms by which *RNase Z* mutations contribute to neuropathology is complicated by its dual role in processing both nuclear- and mitochondrial-encoded pre-tRNA molecules. While the organelle-specific contributions to disease etiology and progression remained uncharacterized, multiple studies examined the varying degrees of damage that disease-related mutations bring upon nuclear and mitochondrial tRNA processing. Importantly, the two substrates of RNase Z cleavage are very distinct – nuclear-encoded tRNAs (nu-tRNA) possess the canonical and rigid cloverleaf structure, while mitochondrial-encoded tRNAs (mt-tRNA), often lacking key structural features such as D or T loops, are termed “aberrant” and “fragile” [51–53]. Despite these structural differences, wild-type RNase Z is capable of recognizing and processing both substrates. Indeed, inactivation of RNase Z, either via knockout or RNAi, leads to the accumulation of nu-tRNA and mt-tRNA processing intermediates [12,16,18,22,28]. Structural analyses from Cryo-EM studies propose two distinct modes of substrate recognition: RNase Z directly binds canonical nu-tRNA, whereas mt-tRNA processing requires the TRM10C/SDR5C1 complex as a scaffold for proper 3′-end cleavage [54]. Although the model implicating additional proteins in the processing of non-canonical substrates is compelling, an alternative model argues that RNase Z is entirely self-sufficient. That is, independently of whether the substrate is canonical or not, the enzyme employs its N-terminal flexible arm to recruit pre-tRNAs and guides the 3′ trailer into the catalytic site via its C-terminal helix domain [55]. While the mechanistic details of RNase Z catalysis remain to be resolved, *in vitro* cleavage assays have revealed that the enzyme’s catalytic efficiency varies depending on substrate type. In particular, the specificity constant (k_cat_/K_m_) is consistently higher for nu-tRNAs than mt-tRNAs, indicating that mitochondrial pre-tRNAs are intrinsically less favorable substrates [56,57]. Furthermore, several pathogenic RNase Z variants identified in human disorders are unable to cleave mitochondrial pre-tRNAs while retaining the ability to process nuclear pre-tRNAs [7,11,58]. *In vivo* analyses similarly indicate that some disease-associated RNase Z alleles cause pronounced mitochondrial dysfunction, while no nuclear damage is noted [7,22]. Altogether, these data suggest that phenotypes associated with certain missense substitutions in RNase Z could be mitochondrially based.

Indeed, here, we found that when neuronal cells are completely deprived of or carrying the Thr494Ile mutant form of RNase Z, mitochondria become dysfunctional. They undergo fragmentation, change shape from tubular to globular, and neurons accumulate ROS. To isolate organelle-specific contribution of mitochondria to RNase Z-associated neuropathology, we generated two strains of flies that carry genetically modified RNase Z only in mitochondria of neuronal cells – the KO (*Elav-Cas9 > mt-RNZ*^*KO*^) and the mutant strain (*Elav-Cas9 > mt-RNZ*^*T494I*^). The longevity of corresponding adults was reduced to a similar degree as was found in flies with pan-neuronal RNase Z modification. Flies without RNase Z or mt-RNase Z in their neurons did not survive past two days after eclosion. Flies with the Thr494Ile variant of RNase Z expressed pan-neuronally or only in mitochondria had a median lifespan reduced to ~35 days. It is noteworthy that cellular and organelle-specific perturbations of neuronal RNase Z produce the same extent of damage to the lifetime of adults.

Testing flies harboring the mutant form of mitochondrial RNase Z^T494I^ in neurons, we found that both locomotor activity and associative learning ability were impaired. Importantly, the extent of impairment was very similar in pan-neuronal and mitochondria-specific mutants. Both the *Elav-Cas9 > RNZ*^*T494I*^ and *Elav-Cas9 > mt-RNZ*^*T494I*^ flies displayed a reduction in flight ability. Motoneurons at the NMJ and KCs of the MB had aberrant structure and activity in the *Elav-Cas9 > RNZ*^*T494I*^ and *Elav-Cas9 > mt-RNZ*^*T494I*^ flies. Altogether, our data show that replacing wild-type RNase Z with the Thr494Ile variant in the neuronal mitochondria is sufficient to impair motor function and learning/memory ability. Although direct pathogenic mechanisms remain to be elucidated, our data show that the presence of the Thr494Ile form of RNase Z in the neuronal mitochondria is sufficient to impair motor function and learning/memory ability. This implies that substitution of a highly conserved amino acid in RNase Z diminishes its enzymatic activity on aberrant mt-tRNAs, conferring functional damage to mitochondria and contributing to the observed neuropathology.

Consistent with our findings, other studies have shown that some neurological conditions are triggered by mitochondrial impairment, further leading to impaired locomotion and learning/memory [59,60]. Given the central role of mitochondria in aerobic ATP production, tissues with high energy demands, such as neurons, are highly sensitive to mitochondrial dysfunction. During fly metamorphosis, many larval neurons undergo dramatic remodeling. Select motor neurons, sensory neurons, and KCs of the MB prune their neurites, then regrow and reestablish synaptic connectivity within the adult nervous system. This transition is heavily dependent on mitochondria-efficient energy production via OXPHOS [61–64]. In this study, we observed elevated ROS and fragmented mitochondria in neurons deprived of RNase Z, suggesting that damaged mitochondria may negatively affect neurodevelopment of the adult MB and motor neurons at the NMJ.

Besides energy production, mitochondria participate in many other cellular functions. In neurons, they regulate local calcium (Ca^2+^) levels, which are critical for neuronal excitability and synaptic transmission [65]. Previous studies have shown that fragmentation hinders the efficient transmission of Ca^2+^ between mitochondria and ER [66], which may affect synaptic function [67]. Indeed, in our experiments, we found that, in the presence of the mutant form of RNase Z, mitochondria undergo increased fission, and synaptic transmission is delayed or even breaks down during periods of high-frequency stimulation.

In addition to being involved in the synthesis of mature tRNA molecules, RNase Z also aids in the generation of tRNA-derived fragments (tRFs). These small non-coding RNAs are known to regulate gene expression, metabolism, and apoptosis [68]. Specifically, RNase Z has been implicated in the production of mitochondrial tRFs, one of which has been recently identified to interact with ETC complexes and regulate mitochondrial respiration [69,70]. It is plausible that RNase Z^T494I^ alters mt-tRF production, eventually leading to mitochondrial dysfunction. Additional research is required to explore this mechanism.

Overall, our studies show that mutating neuronal mt-RNase Z is sufficient to induce neuropathogenesis. The Thr494Ile allele affects energy-demanding neurons, recapitulating symptoms frequently observed in human patients.

### Concluding remarks

The major accomplishment of the current study is the generation of a fly model of *ELAC2*-associated human neuropathology. Our *in vivo* analysis provides foundational evidence that the Thr494Ile variant of RNase Z is pathogenic in neurons, particularly in neuronal mitochondria. A single amino acid substitution, introduced into neuronal mt-RNase Z, is sufficient to recapitulate *ELAC2*-associated human disease. At the same time, we acknowledge that our study has limitations. The underlying cellular/molecular mechanisms causing locomotor deficit and learning/memory disability, among other neurological phenotypes in *Elav-Cas9 > RNZ*^*T494I*^ flies, remain unresolved. While we identified mt-RNase Z as a primary contributor to disease and a cause of functional damage to mitochondria, it is unclear whether the various neuronal phenotypes we report arise from a unified mechanism, specifically, mitochondrial dysfunction, or from distinct, perhaps parallel processes. These mechanistic gaps at the molecular level warrant further investigation in the future. Those may include testing the impact of ROS scavengers on phenotypic rescue, performing transcriptomic analyses to identify altered pathways, and conducting genetic interaction studies with regulators of mitochondrial dynamics and function (*e.g.*, Marf, Complex I subunits).

## Materials and methods

### Fly stocks

Flies were maintained at 25°C on standard cornmeal-molasses-agar medium unless specified. The following flies from the Drosophila Stock Center (Bloomington, IN, USA) were used in this study: *elav-Gal4* (BDSC #458) and *UAS-mitoDsRed* (BDSC #93056).

The transgenic lines carrying U6-3xgRNA^Z^, gZ^+^-PAM^Δ^-V5 (*RNZ*^*+*^), and gZ^T494I^-PAM^Δ^-V5 (*RNZ*^*T494I*^) constructs were established and described previously [32]. The transgene encoding a Cas9-resistant form of RNase Z lacking mitochondrial targeting sequence (*mt-RNZ*) was generated by sequentially introducing single-nucleotide substitutions into each of the three PAM sites within the *genRNZ*^*∆MTS*^*-V5* expression construct, which was created previously [16]. Transgenic lines were generated by phiC31-mediated integration into the attP site on the third chromosome at 68A4 (GenetiVision). Using Western blot analysis, we confirmed stable expression of the RNase Z variants in respective transgenic lines (S10 Fig).

The *elav-Cas9* transgene was generated by introducing the *elav* enhancer from the p{ELAV-GeneSwitch} plasmid (Addgene #83957) into the SalI and EcoRV sites of the Gateway pENTR3C vector (ThermoFisher, A10464). This entry plasmid was used in an LR reaction with Gateway Clonase II Enzyme mix (ThermoFisher, 11791020) to transfer the *elav* enhancer into the pDest-APIC-Cas9 vector (Addgene, # 121657). Transgenic lines were generated by phiC31-mediated integration into the attP site on the second chromosome at 57F5 (GenetiVision). Transgene expression was confirmed by Western blot with monoclonal anti-Cas9 antibodies (1:1000, Invitrogen, MA5–23519) (S5 Fig).

All experimental models listed in Table 1 were produced via breeding corresponding transgenic parental stocks. To generate animals with neuron specific RNase Z knockout, we crossed *Elav-Cas9* flies with those that ubiquitously express guide-RNA targeting the RNase Z gene (*U6-3xgRNA*^*Z*^) and studied their offspring (*Elav-Cas9 > RNZ*^*KO*^). Animals that harbor the mutant variant of RNase Z (*Elav-Cas9 > RNZ*^*T494I*^) in neurons were produced in the cross between *Elav-Cas9* and *U6-3xgRNA*^*Z*^;*gZ*^*T494I*^*-PAM*^*Δ*^ flies. For neuron-specific knockout of mt-RNase Z (*Elav-Cas9 > mt-RNZ*^*KO*^), we bred *Elav-Cas9* and *U6-3xgRNA*^*Z*^;*gZ*^*ΔMTS*^*-PAM*^*Δ*^ flies. Flies deprived of neuronal mt-RNase Z and rescued with the Thr494Ile variant (*Elav-Cas9 > mt-RNZ*^*T494I*^) were generated in the cross between *elav-Cas9;gZ*^*T494I*^*-PAM*^*Δ*^ and *U6-3xgRNA*^*Z*^;*gZ*^*ΔMTS*^*-PAM*^*Δ*^ flies. Control flies (*Elav-Cas9 > RNZ*^*+*^) were produced in the cross between *Elav-Cas9* and *U6-3xgRNA*^*Z*^;*gZ*^*+*^*-PAM*^*Δ*^ flies. The use of *Elav-Cas9 > RNZ⁺* ensures a uniform genetic background among fly lines, making it the most appropriate control for our study.

### Longevity assay

To measure lifespan, flies of the specified genotypes from a synchronized population were collected on the day they eclosed and placed into same-sex cohorts of ten flies per vial. The number of survivors in each vial was scored every other day. The flies were transferred into fresh vials three times per week during the entire test. This assay was continued until every fly died, and the survivorship curve was plotted.

### Negative geotaxis assay

Fly fitness was tested using the negative geotaxis assay. On the day of eclosion, flies from a synchronous population were collected under brief CO_2_ anesthesia (1–2 min) and separated into groups of 10 animals (5 males and 5 females). At all times, the flies were kept on fresh food at 25°C. On the day of analysis, flies were transferred into vials marked with lines forming four equally spaced quadrants. Each vial was gently tapped to bring animals to the bottom of the vial, after which they were given four seconds to climb up before a photograph was taken to record the position of each fly in the vial. Each fly was assigned a rating based on the quadrant it reached; flies that did not climb and stayed at the bottom were scored as zero. The weighted average of three consecutive trials was calculated for each vial and represented a climbing index for that vial.

### Histological analysis for brain vacuolization

Ten flies of the specified genotype were threaded into the collar (Genesee). Specimens were fixed in Carnoy’s fixative (overnight at 4^o^C), then dehydrated using increasing gradients of EtOH solution, washed with xylenes, and placed into hot paraffin. Solidified paraffin blocks were sliced at a transverse orientation at 5 µm thickness. The sections were placed on slides for subsequent hematoxylin and eosin (H&E) staining. (Sigma). All histological samples were analyzed under the Zeiss Axio Imager M1 microscope, and brightfield images were captured with an AxioCam MRc camera. Vacuoles identified by their oval/spheroid shape were selected for area measurements via ImageJ.

### Motor neuron morphology in the flight muscle

This protocol was adapted from Sidisky et al. [71]. Briefly, Dorsal Longitudinal Muscles (DLMs) were dissected by first removing the head and abdomen from the thorax. Thoraces were fixed in 4% paraformaldehyde in 1xPBS for 30 min, washed thrice with 1xPBS for 10 min each, all at RT. Next, samples were flash frozen with liquid nitrogen and bisected down the midline in ice-cold PBS. Tissues were blocked in a buffer (0.1% NGS and 0.2% Triton X-100 in PBS) for 1 hr at 4°C, and then incubated overnight in a dark box at 4°C with goat polyclonal anti-HRP-488 (1:300, Jackson Immunoresearch, 123-545-021) to stain the motor neurons and Texas Red-Phalloidin probe (1:200, Thermo Fisher, T7471) to stain the muscle fibers. The samples were washed with 1xPBS for 3 x 5 min at RT and mounted on slides with Fluoromount antifading medium. A Leica TSP5 laser scanning confocal microscope was used to capture the DLM images with a 63x oil objective by creating a Z-stack of successive slices taken at 0.7 µm intervals. Total neurite length and branch numbers were calculated by tracing neuronal morphology using the Simple Neurite Tracer plug-in in ImageJ [72].

### Giant Fiber electrophysiology

Synaptic transmission was assed following a previously described protocol [33]. Prior to electrophysiological stimulation, the flies were immobilized on ice, fixed to a tungsten pin with cyanoacrylate glue, and then allowed ~15 minutes to recover. Sharpened tungsten stimulation electrodes were inserted into the fly eyes. Another sharpened electrode was inserted into the flight muscle. Lastly, a reference electrode was inserted into the dorsal abdomen. For response latency, an isolated pulse stimulator was used to deliver pulses at 30V for 30 s at a frequency of 2 Hz. For the following frequency, a train of 10 30V stimuli was sequentially provided in increments of 20 Hz, starting from 20 up to 200 Hz. The measurements were recorded by an AC amplifier controlled by a LabVIEW script, and the traces were generated and analyzed using custom-written MATLAB code.

### Flight performance test

Flight performance was measured following a previously described protocol [73]. Flies were collected on the day of eclosion, placed into same-sex cohorts of 20 flies per vial, and aged for 10 and 21 days at 25°C. To initiate flight behavior, vials were dropped in an inverted orientation down a 25 cm drop tube with a funnel attached to the bottom, which stops the vial. The flies continued to fall down into a 90 cm x 13.5 cm measuring tube lined with a removable plastic sheet coated in a sticky Tangle Trap. Fall-triggered flight led to a collision with a tube wall, and flies stuck to the sticky trap. The sheet with flies stuck at various locations was removed, flattened, and photographed. Flies unable to initiate flight (flightless) got caught in a dish of mineral oil, and their landing height was marked as 0. To account for any inadvertent effects of fly handling, we excluded the number of flightless flies equal to that of the age-matched control flies from every genotype studied. The multipoint tool of ImageJ software was used to obtain the y-coordinates of the flies in order to analyze the location of a fly’s landing height. These coordinates were then converted into the landing height to the nearest mark (cm).

### Brain immunostaining

Adult fly brains were dissected in ice-cold 1xPBS and fixed for 20 min at RT in 4% paraformaldehyde. The samples were washed six times for 20 min each and blocked in hybridization buffer (5% BSA, 5% NGS in PBS) for 2 hrs at RT. The brains were incubated with either mouse monoclonal antibody anti-Brp (1:50, DSHB, nc82) or mouse monoclonal antibody anti-Fas2 (1:25, DSHB, 1D4) overnight at 4°C. The samples were washed six times for 20 min each and incubated in secondary goat anti-mouse polyclonal anti-AF647 antibody (1:400, Jackson Immunoresearch, 115-605-062) for 2 hrs at RT. Lastly, the brains were washed in 1xPBS for 3 × 5 min at RT and then mounted on slides with Fluoromount antifading medium. Images were acquired using the Leica TSP5 laser scanning confocal microscope with a 40x objective by creating a Z-stack of successive slices taken at 0.5 µm intervals. The images were then processed, and Max Intensity Projections were obtained using ImageJ. Mushroom body structure was assessed by visually scoring different phenotypes. Aberrant phenotypes were defined as instances of lobe dysmorphology like truncated, overextended, or missing α/α’ lobes and/or fused or missing β/β’ lobes. Thickness of the α/α’ lobe was quantified by measuring the total area and the width using the freehand selection tool in the ImageJ software.

### Olfactory learning assay

Learning and memory assays were carried out using previously established protocols [74,75] with minor modifications. 3–5-day-old flies were trained with aversive or appetitive conditioning protocols using odors 3-octanol (OCT, 1:100) and 4-methylcyclohexanol (MCH, 1:67) diluted in paraffin oil. Memory tests were conducted in a T-maze (CellExplorer Labs) wherein flies could choose between the two arms, each delivering a distinct odor.

For short-term memory (STM) assessment, flies were subjected to aversive conditioning. Briefly, flies were sequentially exposed for 60 s to either odor. The first odor was paired with an electric shock (a conditioned stimulus, or CS+) using a power supply delivering 12 consecutive shocks at 80 V for 1 min. The second odor (CS-) was unpaired. After training and 1-hr recovery period, flies were subjected to the test, when they were choosing between CS+ and CS- odors. The results of the learning assay are presented as a Performance Index (PI). The PI value is calculated as the proportion of flies avoiding CS+ minus fraction of flies avoiding CS- divided by the total number of flies.

For long-term memory (LTM) assessment, flies were trained multiple times with appetitive olfactory conditioning. Prior to training and testing, flies were starved for 16–20 hr by placing them in vials containing 1.5% agar. During training, flies were exposed to the first odor (CS+) in a tube lined with filter paper soaked in 2M Sucrose solution for 1 min. Next, they were exposed to the second odor not paired with the reward (CS-) for another minute. After training, flies were allowed to recover on regular food for 4 hr, then starved and tested. The PI value is calculated as the proportion of flies that choose the CS+ over the CS- divided by the total number of flies.


$$\text{PI }=\frac{\#CS+flies\;-\;\#CS-flies}{\#total\;flies}$$

This assay was repeated 10–12 times, with at least 50 flies per repeat. In half of these repeats, the CS+ and CS- odors were swapped to account for any inherent bias.

### Blue native polyacrylamide gel electrophoresis (BN-PAGE) and In-Gel complex I activity

Mitochondria were purified according to previously published protocol [76] and stored at -80^o^C. BN-PAGE was performed using NativePAGE gels (Life Technologies) per manufacturer’s instructions. Mitochondria were resuspended in NativePAGE Sample Buffer with 1% digitonin and protease inhibitors, incubated on ice for 20 min, then centrifuged at 20,000 × g for 30 min. 25 ul of supernatant was collected, with half the amount used for Western blotting with anti-VDAC (Abcam, 1:1000) antibody (mixed with 2 × Laemmli buffer), and the other half prepared with G-250 Sample Additive and loaded onto 3–12% NativePAGE gels. NativeMark Protein Standard was included to estimate complex sizes. Electrophoresis used NativePAGE Running Buffer (anode) and Running Buffer with 0.4% Coomassie G-250 (cathode). Complex I activity was visualized by incubating gels overnight at room temperature in a solution of 0.1 mg/mL NADH, 2.5 mg/mL nitrotetrazolium blue chloride, and 5 mM Tris-HCl (pH 7.4).

### Mitochondrial morphology assessment

Mitochondrial parameters (number and shape) were assessed by visualizing neuronal mitochondria using *UAS-mitoDsRed* reporter driven by *Elav-Gal4*. Briefly, *Elav-Cas9* was combined with the *Gal4/UAS* driver and reporter elements to generate the *Elav-Gal4;Elav-Cas9;UAS-mitoDsRed* stock. These flies were used to produce animals that lack (*Elav-Gal4;Elav-Cas9/U6-3xgRNA*^*Z*^;*UAS-mitoDsRed*) or harbor the mutant variant of RNase Z (*Elav-Gal4;Elav-Cas9/U6-3xgRNA*^*Z*^;*UAS-mitoDsRed/gZ*^*T494I*^*-PAM*^*∆*^) in neurons. The brains were dissected in ice-cold 1xPBS and mounted on slides with Fluoromount antifading medium for immediate visualization. Images were acquired using the Leica TSP5 laser scanning confocal microscope with a 100x objective by creating a Z-stack of 50 successive slices taken at 0.5 µm intervals. The stacks were then cropped to a Region of Interest (ROI) corresponding to the bifurcation of the α/β lobes. Parameters, such as number, area, and volume, were determined using the 3D Manager Plugin in the ImageJ software. Sphericity (ψ) of mitochondria was calculated using the formula below [60], where Vp is volume of the particle and Sp is surface area of the particle:


$$\psi \;=\frac{\pi^{\text{1}/\text{3}}\;*\;{\left(\text{6}Vp\right)}^{\text{2}/\text{3}}}{Sp}$$

### DHE staining

Reactive oxygen species (ROS) level was evaluated by staining with a fluorescent dye dihydroethidium (DHE), which exhibits an increased intensity of red fluorescence signal upon oxidation in the presence of superoxide radicals [77]. Briefly, adult brains were dissected in Schneider’s medium and incubated with 30 µM DHE in the dark for 20 min at RT. The tissues were then washed 3 times in 1x PBS and mounted on slides with Fluoromount antifading medium for immediate visualization. Images were acquired using the Leica TSP5 laser scanning confocal microscope with a 10x objective by creating a Z-stack of successive slices taken at 1 µm intervals DHE fluorescence was calculated in ImageJ, by subtracting the background fluorescence from the integrated density of the brain, and dividing by the total area of the brain.

### Statistical analysis

All graphs and statistical analyses were performed in GraphPad Prism9. Statistical data are presented as mean ± standard error of the mean (s.e.m). P values for all the comparisons were determined by t-tests or one-way ANOVA, unless specified otherwise. The mean difference was considered statistically significant at the 95% confidence level. Results were considered as not significant (ns) when P > 0.05, significant when 0.01 < P < 0.05 (*), more significant when 0.001 < P < 0.025 (**), very significant when P < 0.001 (***), or extremely significant when P < 0.0001 (****). For longevity analysis, Mantel-Cox test was used. For latency responses, a two-tailed t-test or Mann-Whitney test was used to compare the two genotypes. Figures were assembled with Adobe Photoshop CS2 software (Adobe Systems, San Jose, CA).

## Supporting information

S1 Table Raw experimental data. This file contains the primary data underlying all experiments presented in the figures and graphs of the manuscript. Each worksheet within the file corresponds to a specific experiment.
(XLSX)

S1 File Raw western blots. The summary of raw images of western blots used to present data in S10 Fig.
(PDF)

S1 Fig Schematic illustration of the flight performance test. The flight tester set up involves a drop tube through which vials containing flies are released [73]. When the vial(s) hits the funnel, the flies are ejected into the flight cylinder that houses a 90 cm tall acrylic sheet coated with Tangle-trap. The flies that do not adhere to the sheet pass through the column to a dish below, filled with mineral oil. The sheet is then unrolled, placed on a flat surface, and photographed. Shown on the right is a representative picture of an acrylic sheet, where each dot represents the location of an individual fly. The captured image is subsequently used to measure the landing height of each fly using ImageJ.
(TIF)

S2 Fig Neuron-specific RNase Z KOs result in reduced brain size in larvae and adults. (A) Representative brightfield images of third instar larval brains of 5d AED control *Elav-Cas9 > RNZ*^*+*^, 5d AED, and 6d AED *Elav-Cas9 > RNZ*^*KO*^ (n = 10, for all groups). Scale bar represents 100 µm. (B) Quantification of average larval brain lobe volume from animals analyzed in A-C. ****p < 0.0001 (One-way ANOVA followed by Dunnett’s multiple comparison test). Representative confocal images of Hoechst-stained 1-day-old (C) control *Elav-Cas9 > RNZ*^*+*^ and neuronal KO *Elav-Cas9 > RNZ*^*KO*^ (n = 10 for both groups). The scale bar represents 100 µm. Quantification of adult brain size by measuring (D) total brain area and (E) brain width. ****p < 0.0001 (unpaired Student’s t-test). Error bars indicate the mean ± SD.
(TIF)

S3 Fig Loss of neuronal RNase Z affects synaptic architecture in the adult fly brain. Representative confocal images of 1-day-old adult brains stained with the nc82 monoclonal antibody (magenta) raised against the synaptic protein Bruchpilot (anti-Brp). The top panels are the control flies (*Elav-Cas9 > RNZ*^*+*^) and the bottom panels are the neuron-specific KO flies (*Elav-Cas9 > RNZ*^*KO*^). Arrows indicate the neurite tracts surrounding the mushroom bodies.
(TIF)

S4 Fig Mushroom body morphology during different stages of development in flies deprived of neuronal RNase Z. Representative images of brains of control (*Elav-Cas9 > RNZ*^*+*^) and mutant (*Elav-Cas9 > RNZ*^*KO*^) flies stained with anti-FasII. Each panel shows the MB morphology observed at different stages of development.
(TIF)

S5 Fig Schematic illustration of the olfactory learning assays. The learning/memory capabilities are measured by subjecting flies to a training and testing paradigm involving a T-maze. The top panel represents the appetitive conditioning used to assay for long-term memory, where the training involved pairing the first odor with a sugar reward and a second odor without. The bottom panel represents the aversive conditioning used to assay for short-term memory, where the presenting electric shock with the first odor, and the second odor without. The testing phase is identical to both paradigms. After resting, the flies are then kept in the middle chamber and subjected to both odors simultaneously. The flies trapped in the two tubes are collected and used to calculate the performance index.
(TIF)

S6 Fig Flies do not inherently prefer either odor. Control (*Elav-Cas9 > RNZ*^*+*^) and mutant (*Elav-Cas9 > RNZ*^*T494I*^ or *Elav-Cas9 > mt-RNZ*^*T494I*^) flies do not prefer OCT or MCH when presented with both odors in the absence of training. Preference for either odor was assessed using Performance Index (PI) values, which were measured for every set of flies tested. Each dot represents a PI value calculated from a single trial (n > 500). ns., not significant (unpaired student’s t-test). Error bars indicate the mean ± SD.
(TIF)

S7 Fig Mitochondrial Complex I activity in control and mutant flies. (A) The top panel shows the in-gel CI activity of Z^24^;RNZ^+^, Z^24^;RNZ^T494I^ and Mef2 > RNZi. The bottom panel shows the Western blot with anti-VDAC antibodies serving as a loading control. (B) Quantification of the relative band intensity normalized to the loading control as seen in (A) (n = 3). *p < 0.0332, **p < 0.0021, ***p < 0.0002, and ****p < 0.0001 (unpaired student’s t-test).
(TIF)

S8 Fig Schematic of RNase Z protein variants encoded by respective rescue transgenes. *Drosophila* RNase Z has a Mitochondrial Targeting Sequence (MTS) sandwiched between two alternative translation-initiating methionines - Met^1^ and Met^24^. One Nuclear Localization Signal (NLS) ^759^RKRK^762^ is at the carboxy end. Amino-terminal sequences are shown for the wild-type and the ΔMTS variants of RNase Z. Initiating methionines are in red, the mutated Leu (former Met^1^) is underlined, V5-tags are attached to the carboxy-termini.
(TIF)

S9 Fig Flies deprived of neuronal mt-RNase Z and rescued with the RNase Z^T494I^ variant. (A) Survival rates for control *Elav-Cas9 > RNZ*^*+*^ and mutant *Elav-Cas9 > mt-RNZ*^*T494I*^ flies (n = 100 for each genotype). ****P < 0.0001 (Mantel-Cox test). (B) Negative geotaxis expressed as a climbing index is shown for control and mutant flies at 10 and 30 days of age (n > 100 for each genotype). ****p < 0.0001 (unpaired student’s t-test). Error bars indicate mean ± SD.
(TIF)

S10 Fig Analysis of transgenic RNase Z expression. Western blot analysis of the RNase Z proteins encoded by the corresponding transgenes. Expression of all proteins is driven by the natural RNase Z promoter [18]. The RNZ^+^-PAM^+^-V5 flies are those that do not harbor any of the single-nucleotide replacements in the PAM sequence. The *white* (*w*^*1118*^) stock flies are used as a negative control; α-Tubulin is a loading control. RNase Z is detected with the anti-V5 antibody.
(TIF)

S11 Fig Verification of Cas9 expression. Western blot analysis of Cas9 proteins whose expression is driven by indicated promoters. The *white* (*w*^*1118*^) stock flies are used as a negative control; α-Tubulin is a loading control. Cas9 expression is detected with the anti-Cas9 antibody.
(TIF))
