# Supplementary material for: Mitochondrial-specific perturbation of Drosophila RNase Z in neurons leads to motor impairments, disrupted learning and neurodegeneration
Source: PLoS Genet. 2025 Nov 3;21(11):e1011938. doi: 10.1371/journal.pgen.1011938 (PMC12614798; doi:10.1371/journal.pgen.1011938)
Supplement: S1 File — The summary of raw images of western blots used to present data in S10 Fig. (PDF) [file pgen.1011938.s002.pdf]

Mitochondrial-specific perturbation of Drosophila RNase Z in neurons leads to motor impairments, disrupted learning and neurodegeneration

Raw images of the Western blot

<u>Figure S10:</u> <u>Analysis of transgenic RNase Z expression.</u>

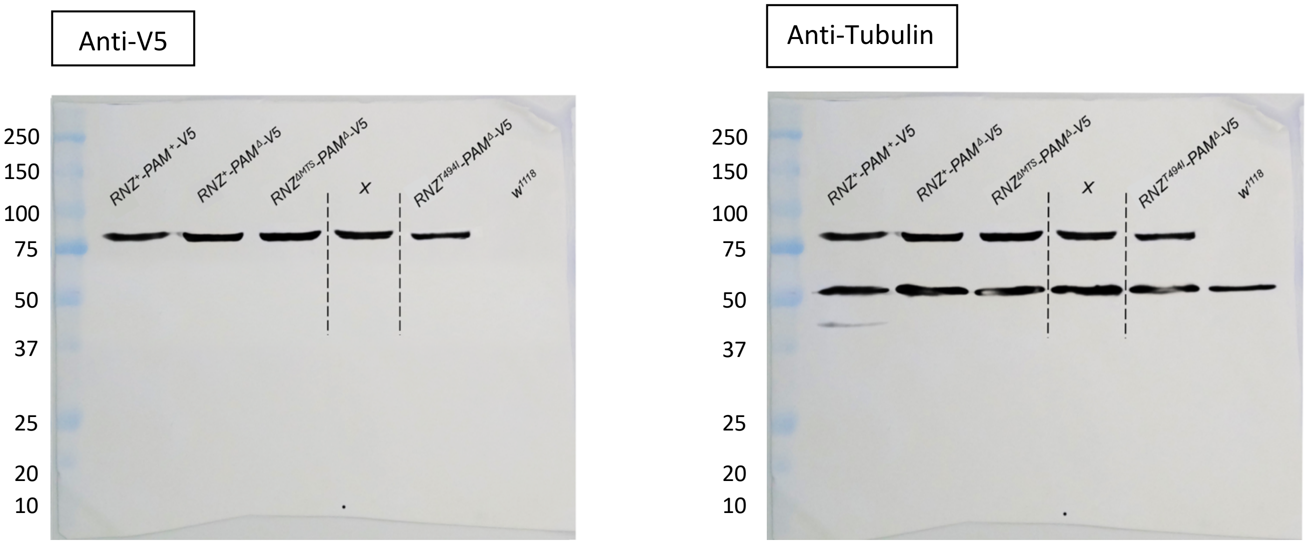

Raw images of blots pertaining to figure S10. The white1118 stock flies are used as a negative control; α-Tubulin is a loading control. RNase Z is detected with the anti-V5 antibody. The fifth lane contains a sample that is not covered in this manuscript and hence spliced out from the final figure. The bands from the anti-V5 blot, and the lower bands from the anti-tubulin blot were used for the final image.
