# Supplementary figures and images for: Mitochondrial-specific perturbation of Drosophila RNase Z in neurons leads to motor impairments, disrupted learning and neurodegeneration
Source: PLoS Genet. 2025 Nov 3;21(11):e1011938. doi: 10.1371/journal.pgen.1011938 (PMC12614798; doi:10.1371/journal.pgen.1011938)

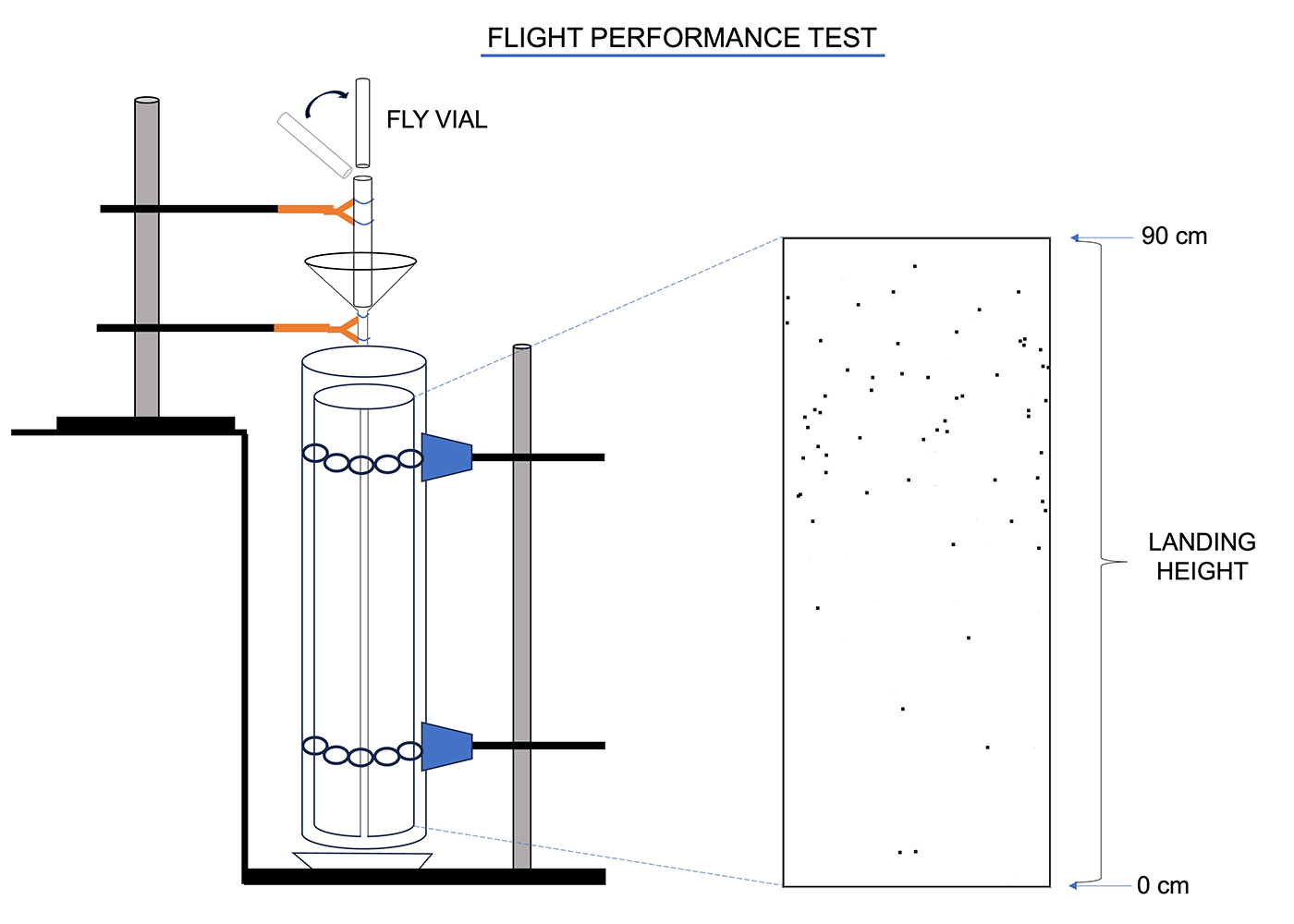

Supplement: S1 Fig — The flight tester set up involves a drop tube through which vials containing flies are released [73]. When the vial(s) hits the funnel, the flies are ejected into the flight cylinder that houses a 90 cm tall acrylic sheet coated with Tangle-trap. The flies that do not adhere to the sheet pass through the column to a dish below, filled with mineral oil. The sheet is then unrolled, placed on a flat surface, and photographed. Shown on the right is a representative picture of an acrylic sheet, where each dot represents the location of an individual fly. The captured image is subsequently used to measure the landing height of each fly using ImageJ. (TIF) [file pgen.1011938.s003.tif]

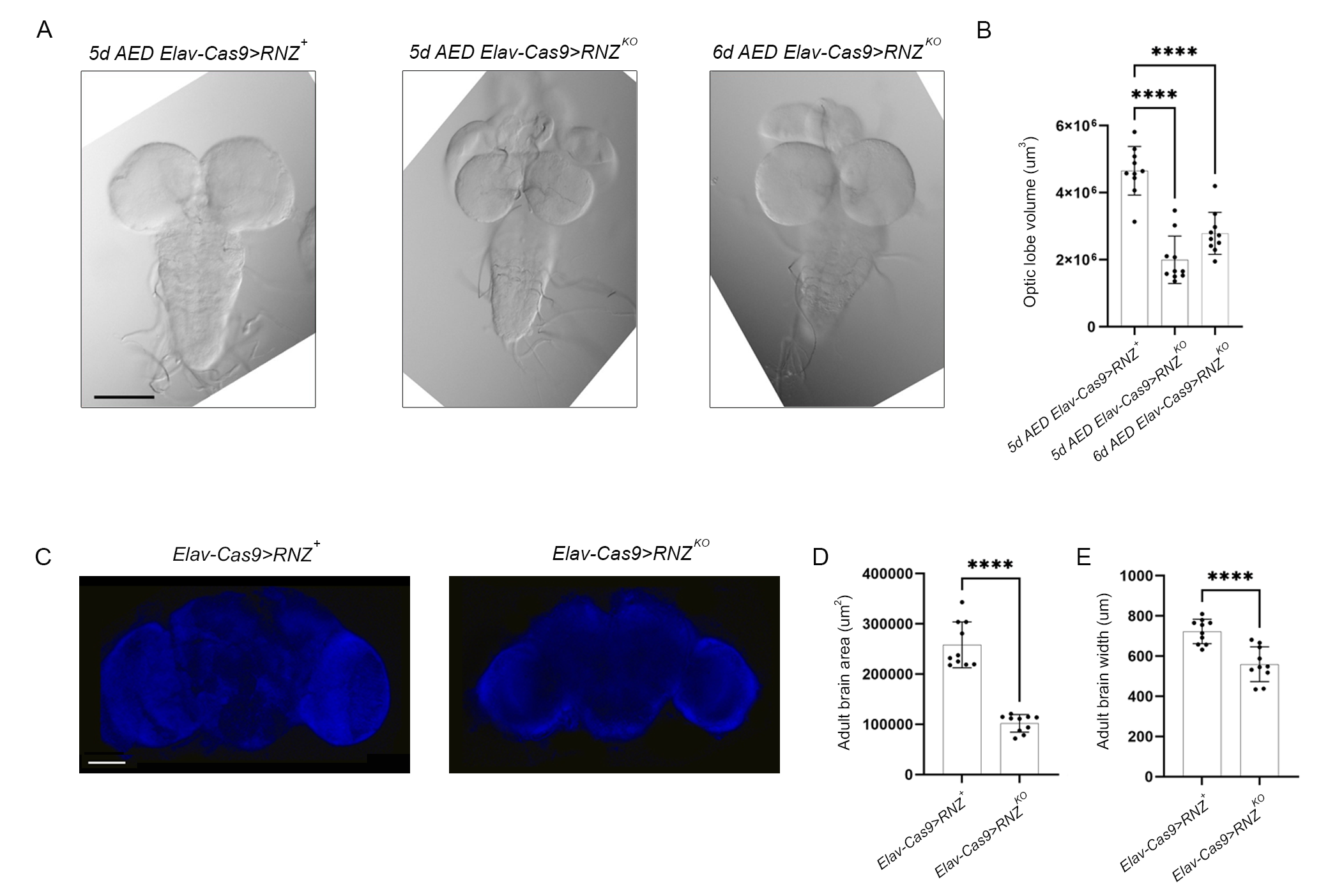

Supplement: S2 Fig — (A) Representative brightfield images of third instar larval brains of 5d AED control Elav-Cas9 > RNZ+, 5d AED, and 6d AED Elav-Cas9 > RNZKO (n = 10, for all groups). Scale bar represents 100 µm. (B) Quantification of average larval brain lobe volume from animals analyzed in A-C. ****p < 0.0001 (One-way ANOVA followed by Dunnett’s multiple comparison test). Representative confocal images of Hoechst-stained 1-day-old (C) control Elav-Cas9 > RNZ+ and neuronal KO Elav-Cas9 > RNZKO (n = 10 for both groups). The scale bar represents 100 µm. Quantification of adult brain size by measuring (D) total brain area and (E) brain width. ****p < 0.0001 (unpaired Student’s t-test). Error bars indicate the mean ± SD. (TIF) [file pgen.1011938.s004.tif]

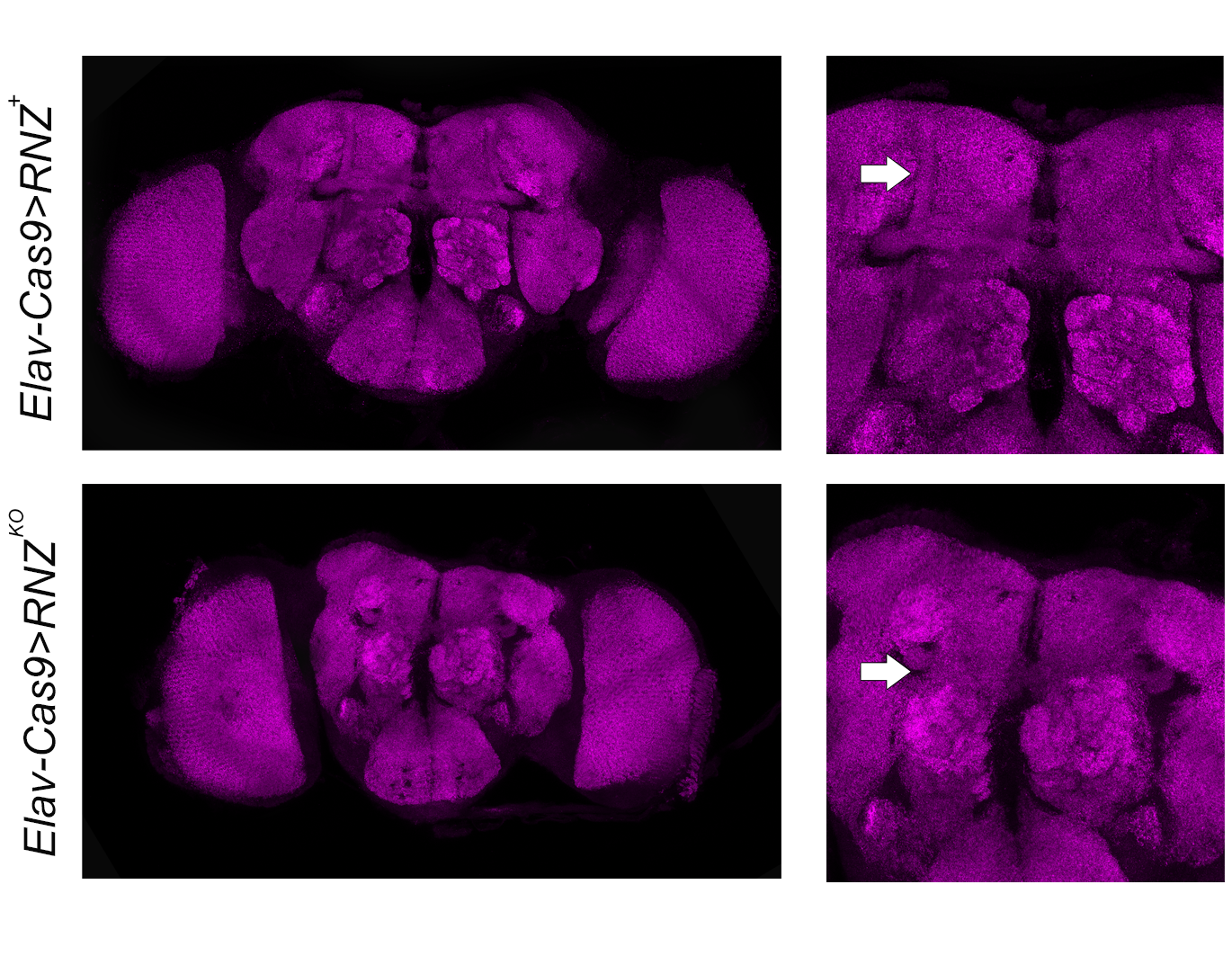

Supplement: S3 Fig — Representative confocal images of 1-day-old adult brains stained with the nc82 monoclonal antibody (magenta) raised against the synaptic protein Bruchpilot (anti-Brp). The top panels are the control flies (Elav-Cas9 > RNZ+) and the bottom panels are the neuron-specific KO flies (Elav-Cas9 > RNZKO). Arrows indicate the neurite tracts surrounding the mushroom bodies. (TIF) [file pgen.1011938.s005.tif]

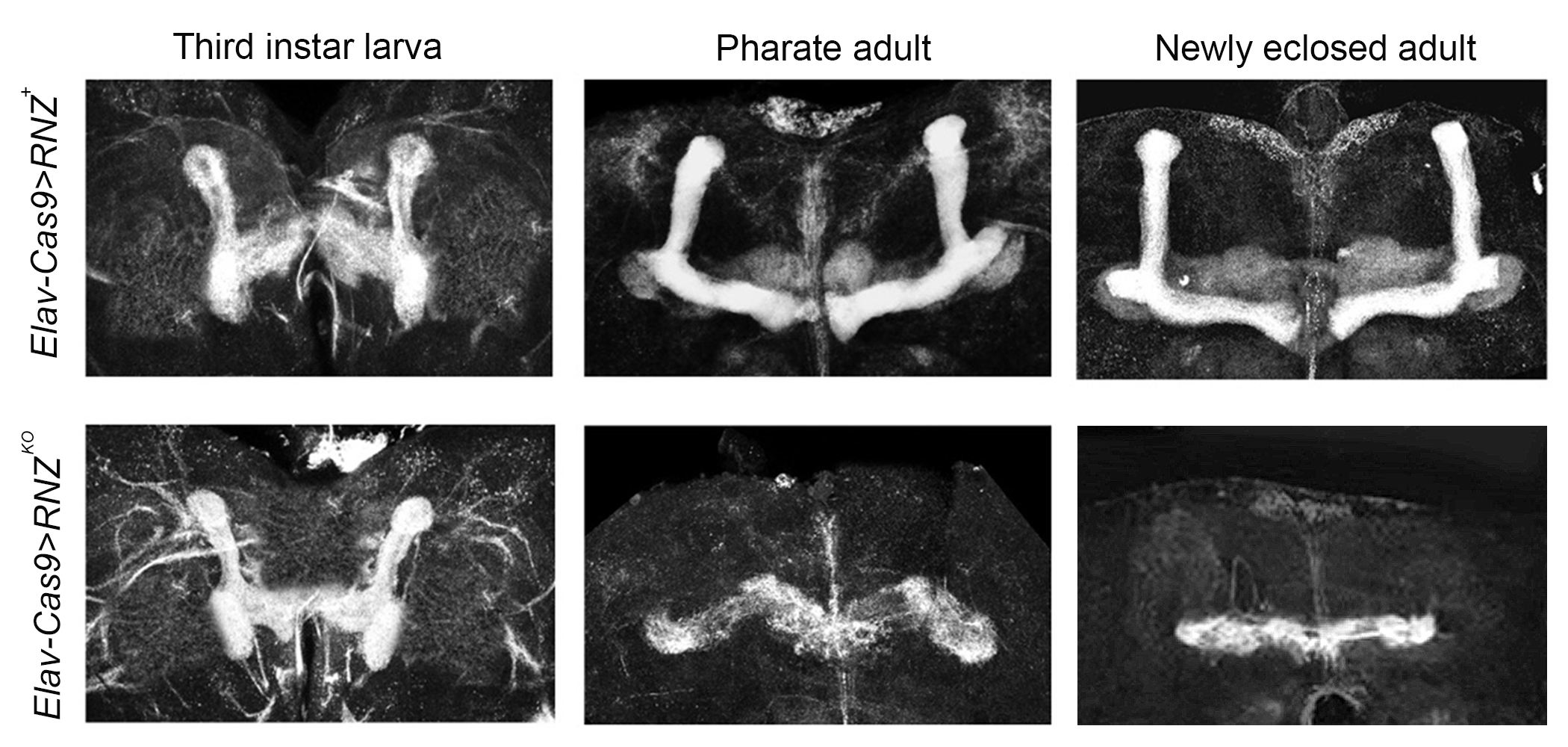

Supplement: S4 Fig — Representative images of brains of control (Elav-Cas9 > RNZ+) and mutant (Elav-Cas9 > RNZKO) flies stained with anti-FasII. Each panel shows the MB morphology observed at different stages of development. (TIF) [file pgen.1011938.s006.tif]

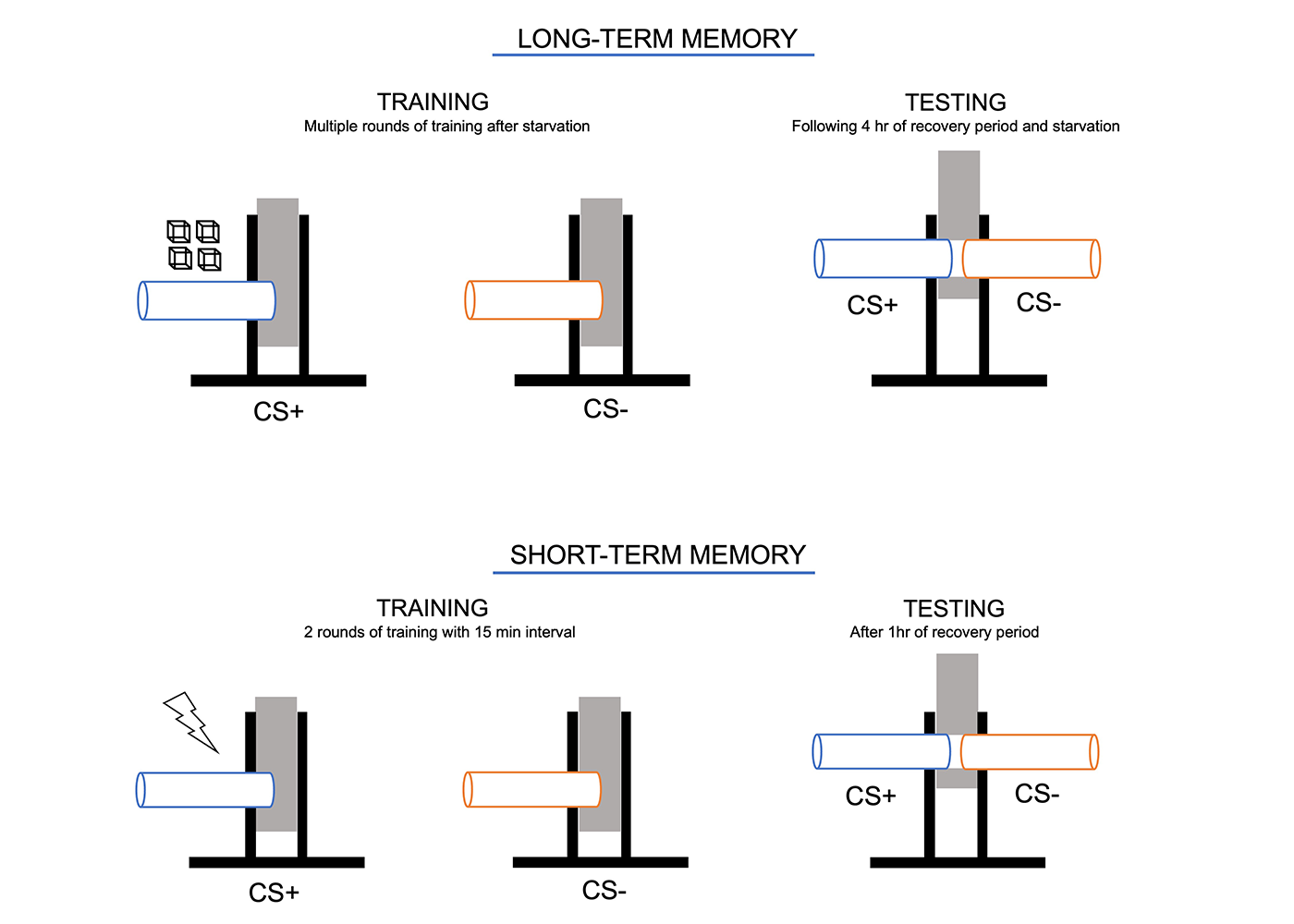

Supplement: S5 Fig — The learning/memory capabilities are measured by subjecting flies to a training and testing paradigm involving a T-maze. The top panel represents the appetitive conditioning used to assay for long-term memory, where the training involved pairing the first odor with a sugar reward and a second odor without. The bottom panel represents the aversive conditioning used to assay for short-term memory, where the presenting electric shock with the first odor, and the second odor without. The testing phase is identical to both paradigms. After resting, the flies are then kept in the middle chamber and subjected to both odors simultaneously. The flies trapped in the two tubes are collected and used to calculate the performance index. (TIF) [file pgen.1011938.s007.tif]

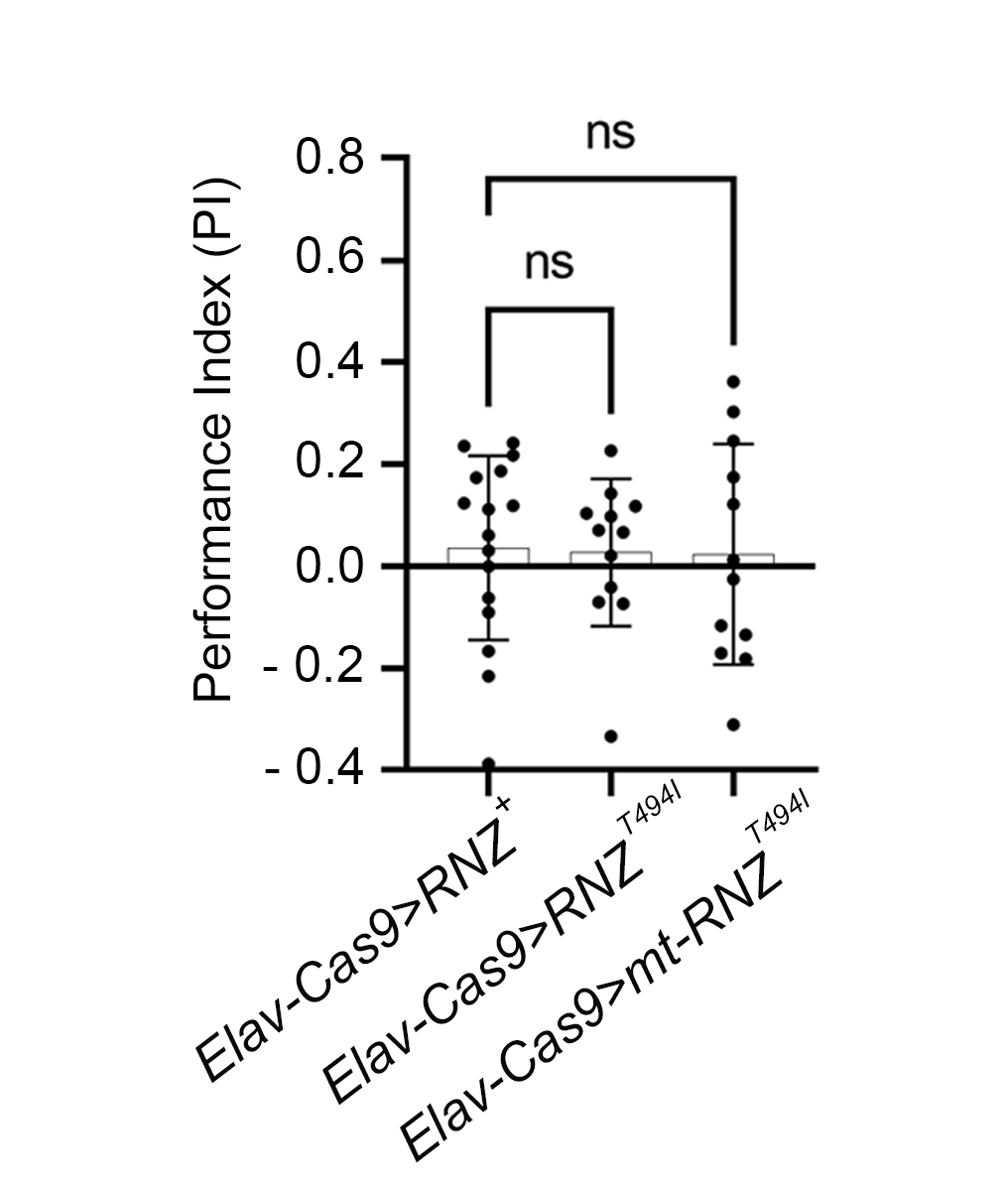

Supplement: S6 Fig — Control (Elav-Cas9 > RNZ+) and mutant (Elav-Cas9 > RNZT494I or Elav-Cas9 > mt-RNZT494I) flies do not prefer OCT or MCH when presented with both odors in the absence of training. Preference for either odor was assessed using Performance Index (PI) values, which were measured for every set of flies tested. Each dot represents a PI value calculated from a single trial (n > 500). ns., not significant (unpaired student’s t-test). Error bars indicate the mean ± SD. (TIF) [file pgen.1011938.s008.tif]

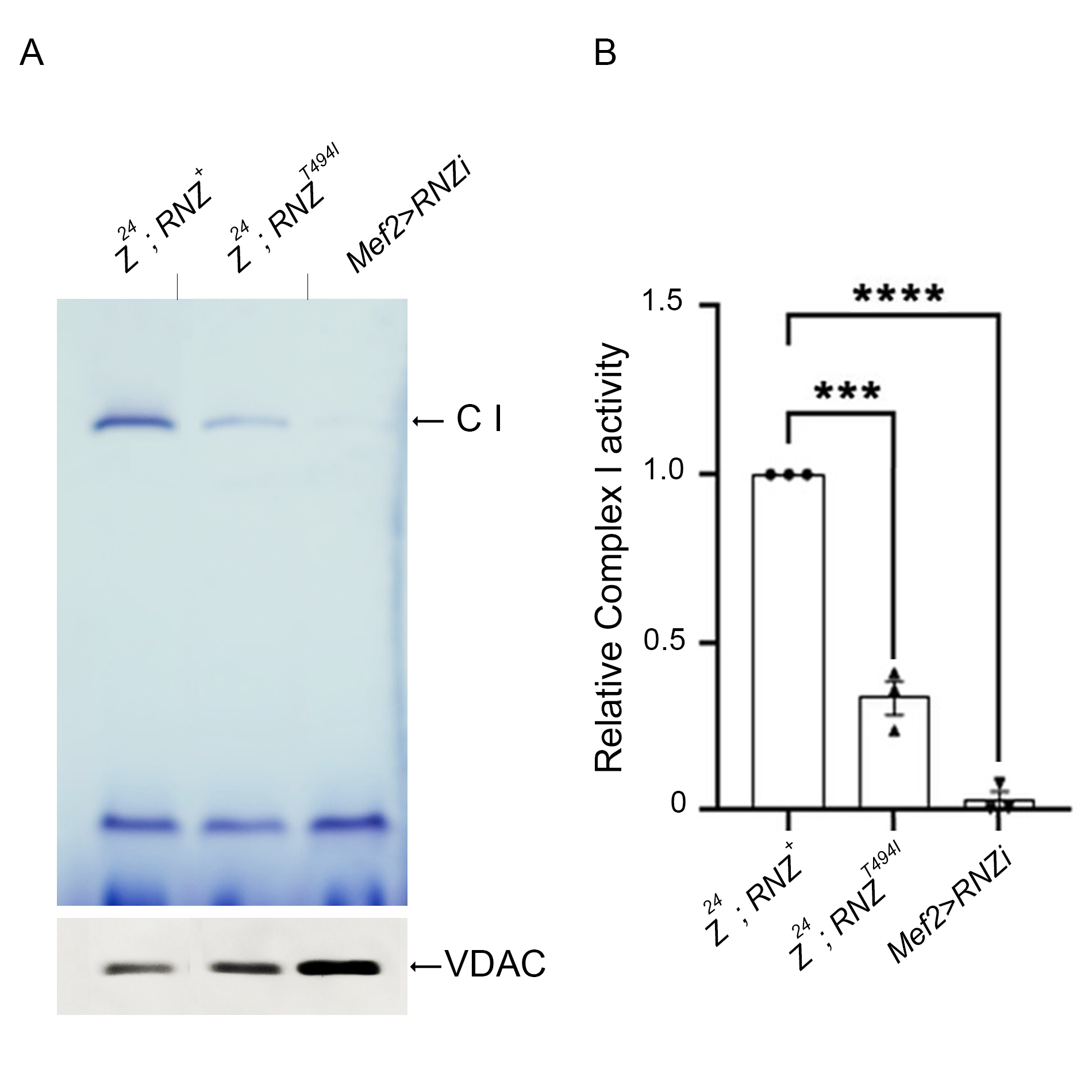

Supplement: S7 Fig — (A) The top panel shows the in-gel CI activity of Z24;RNZ+, Z24;RNZT494I and Mef2 > RNZi. The bottom panel shows the Western blot with anti-VDAC antibodies serving as a loading control. (B) Quantification of the relative band intensity normalized to the loading control as seen in (A) (n = 3). *p < 0.0332, **p < 0.0021, ***p < 0.0002, and ****p < 0.0001 (unpaired student’s t-test). (TIF) [file pgen.1011938.s009.tif]

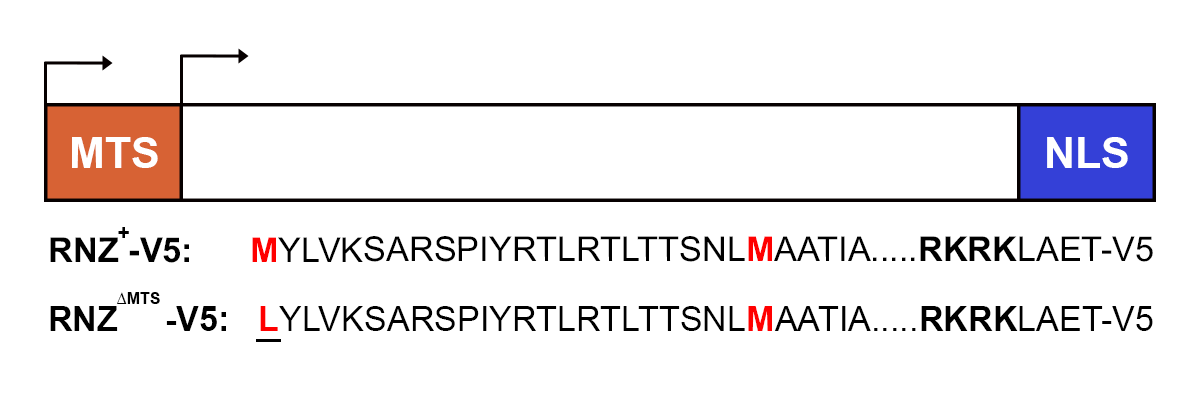

Supplement: S8 Fig — Drosophila RNase Z has a Mitochondrial Targeting Sequence (MTS) sandwiched between two alternative translation-initiating methionines - Met1 and Met24. One Nuclear Localization Signal (NLS) 759RKRK762 is at the carboxy end. Amino-terminal sequences are shown for the wild-type and the ΔMTS variants of RNase Z. Initiating methionines are in red, the mutated Leu (former Met1) is underlined, V5-tags are attached to the carboxy-termini. (TIF) [file pgen.1011938.s010.tif]

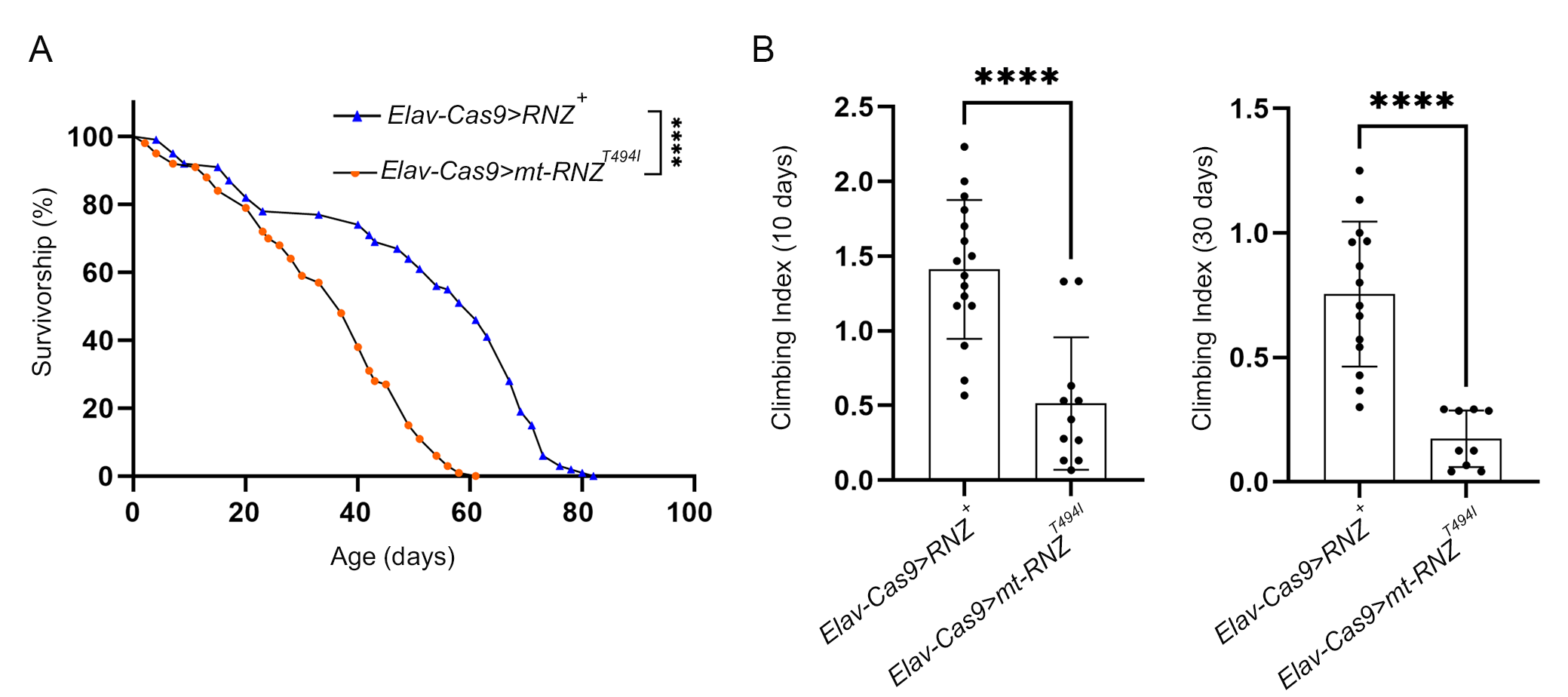

Supplement: S9 Fig — (A) Survival rates for control Elav-Cas9 > RNZ+ and mutant Elav-Cas9 > mt-RNZT494I flies (n = 100 for each genotype). ****P < 0.0001 (Mantel-Cox test). (B) Negative geotaxis expressed as a climbing index is shown for control and mutant flies at 10 and 30 days of age (n > 100 for each genotype). ****p < 0.0001 (unpaired student’s t-test). Error bars indicate mean ± SD. (TIF) [file pgen.1011938.s011.tif]

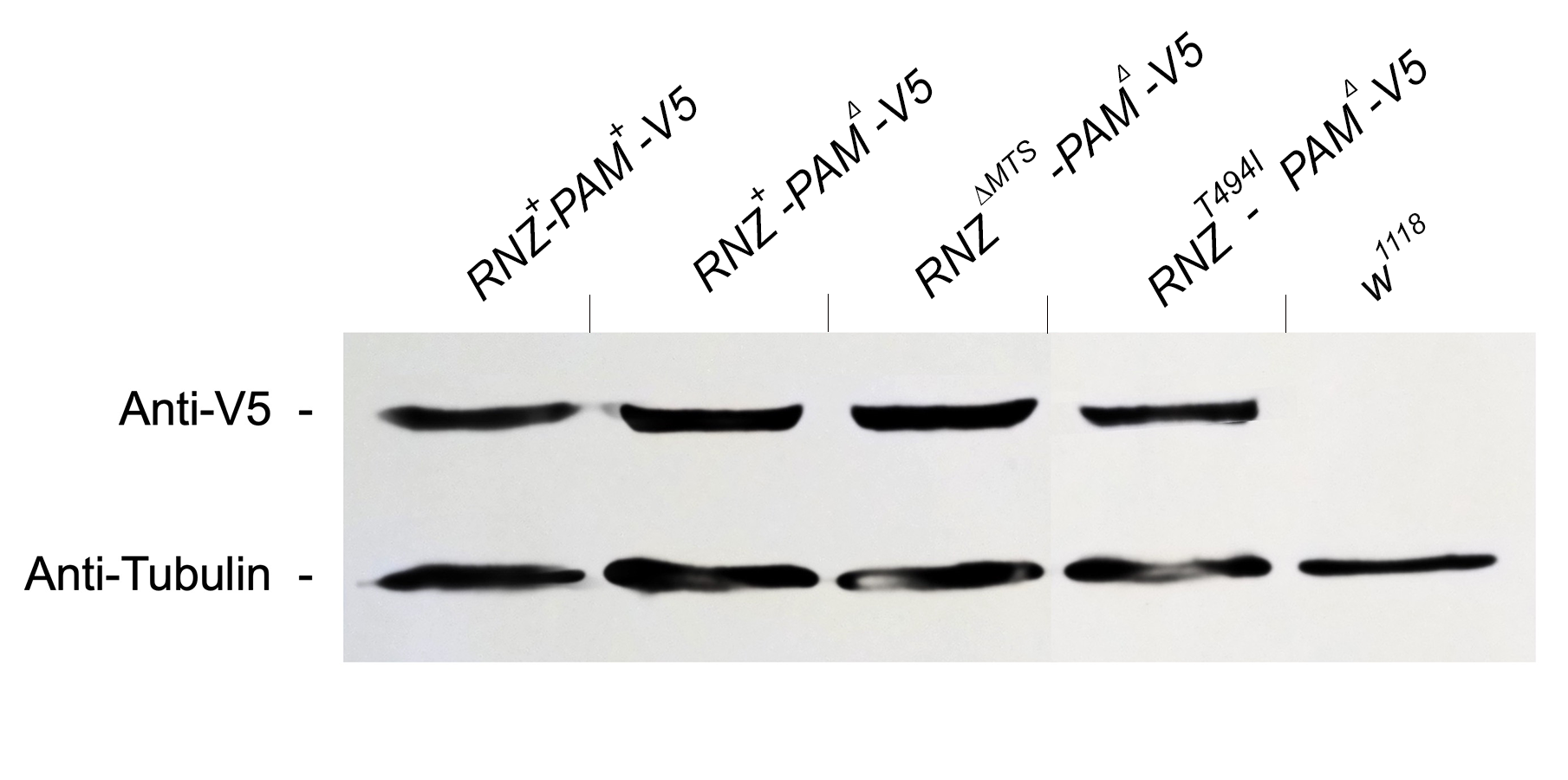

Supplement: S10 Fig — Western blot analysis of the RNase Z proteins encoded by the corresponding transgenes. Expression of all proteins is driven by the natural RNase Z promoter [18]. The RNZ+-PAM+-V5 flies are those that do not harbor any of the single-nucleotide replacements in the PAM sequence. The white (w1118) stock flies are used as a negative control; α-Tubulin is a loading control. RNase Z is detected with the anti-V5 antibody. (TIF) [file pgen.1011938.s012.tif]

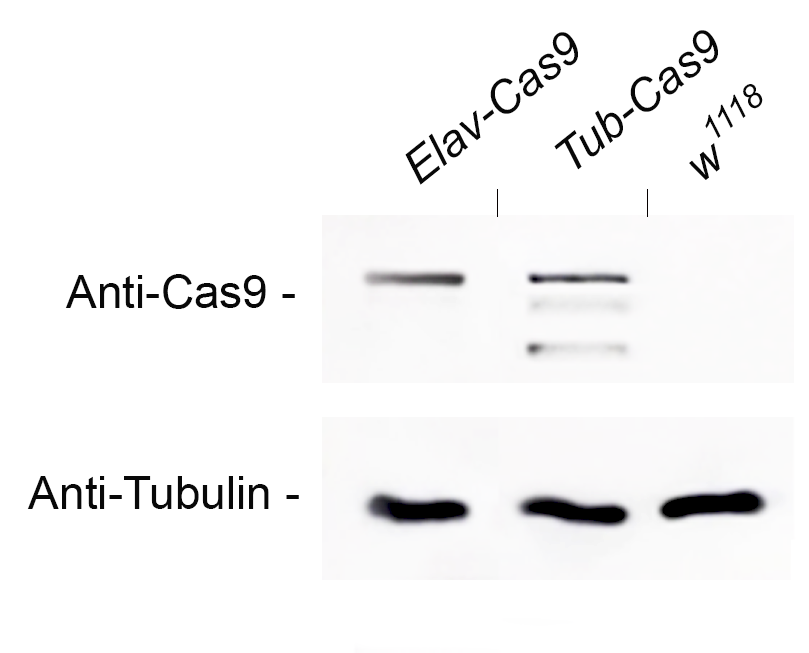

Supplement: S11 Fig — Western blot analysis of Cas9 proteins whose expression is driven by indicated promoters. The white (w1118) stock flies are used as a negative control; α-Tubulin is a loading control. Cas9 expression is detected with the anti-Cas9 antibody. (TIF)) [file pgen.1011938.s013.tif]
